# Machine‐Learning Decomposition Identifies a Big Two Structure in Human Personality with Distinct Neurocognitive Profiles

**DOI:** 10.1002/advs.202509009

**Published:** 2026-03-25

**Authors:** Kaixiang Zhuang, Ji Chen, Jinfeng Han, Wei Cheng, Jiang Qiu, Jianfeng Feng, Simon Eickhoff, Deniz Vatansever

**Affiliations:** ^1^ Institute of Science and Technology for Brain‐Inspired Intelligence Fudan University Shanghai China; ^2^ Center for Brain Health and Brain Technology Global Institute of Future Technology Shanghai Jiao Tong University Shanghai China; ^3^ School of Psychology Southwest University Chongqing China; ^4^ Institute of Neuroscience and Medicine Brain and Behaviour (INM‐7) Research Center Jülich Jülich Germany; ^5^ Institute of Systems Neuroscience Medical Faculty Heinrich Heine University Düsseldorf Düsseldorf Germany

**Keywords:** big two, machine learning, neurocognitive profiles, personality traits

## Abstract

Personality neuroscience has traditionally relied on the Big Five model to investigate trait structure and its relationship to individual differences in brain organization and life outcomes. However, existing theoretical frameworks explain only part of the item‐level covariance, raising questions about whether alternative factor solutions might complement the canonical five‐factor model. Here, we applied an additive and part‐based machine learning decomposition to a mega‐scale, global dataset (*n* = 1,336,840) to systematically evaluate trait structure across factor resolutions. Beyond reproducing the canonical Big Five, we identified a robust Big Two comprising Social Adaptation and Spontaneous Mentation. Social Adaptation integrates covarying questionnaire items from Extraversion, Agreeableness, and Conscientiousness, indexing externally oriented social functioning. Spontaneous Mentation, in turn, aggregates Neuroticism with introspective facets of Openness, capturing internally directed affective‐cognitive exploration. Embedding individuals in this Big Two space revealed structured manifolds along which neurocognitive profiles aligned with distinct trait orientations. Importantly, this lower‐dimensional representation improved prediction of functional brain connectivity relative to Big Five scores, while preserving comparable associations with cognition and mental health. Together, these results establish a neurocognitively grounded Big Two framework that complements the Big Five and offers an interpretable bridge between personality structure, cognitive functioning, and psychopathology.

## Graphical abstract

Using machine learning on a mega‐scale global dataset (*n* = 1,336,840) reveals a robust personality trait architecture beyond the Big Five. A Big Two model, broadly capturing social engagement and internal mentation, defines a geometric space that links personality to neurocognitive profiles. This framework bridges personality theory and neurobiology, offering fresh insights into human behavior and mental health.

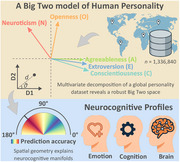

## 1 Introduction

As a fundamental construct within psychological sciences, personality encompasses stable and consistent dispositions of individuals that reflect enduring aspects of human cognition and behavior [1]. Among the most influential theories of personality, the five‐factor model (FFM), also known as the Big Five [2], has gained notable recognition for its capacity to predict life outcomes, including social relationships, career success, educational attainment, mental health [3] and even large‐scale organizational features of the human brain [4].

Despite its widespread adoption, the optimal level of factor resolution for describing personality structures within the Big Five framework remains a matter of debate. While several studies advocate for finer‐grained decompositions that increase dimensional specificity [5, 6], others emphasize the need for alternative representations that capture underlying regularities across the existing trait dimensions [7]. Among these lower‐dimensional formulations, two‐factor models have gained particular prominence in both questionnaire‐based and psycholexical research. For example, higher‐order analyses of the shared variance between Big Five personality traits have consistently revealed two broad dimensions, such as Stability and Plasticity [8, 9]. Similarly, factor‐analytic studies that decompose large sets of item‐level personality descriptors have also identified an analogous configuration, largely defined by Communion/Social Self‐Regulation and Agency/Dynamism, as cross‐culturally recurring, broad dimensions of personality [10, 11]. Although these two‐factor formulations employ different methodological approaches and vary in their specific trait compositions, they exhibit clear conceptual parallels, each contrasting socially oriented self‐regulation with more dynamic, agentic forms of behavior.

In addition to providing a parsimonious framework, such low‐dimensional models offer several other advantages. They can provide a conceptual and empirical bridge between personality science and adjacent domains such as psychopathology and cognitive function, where broad, transdiagnostic factors of vulnerability or adaptation cut across specific symptom patterns and cognitive processes [12, 13]. This integrative potential is further supported by findings of substantial genetic and neural covariance observed across fine‐grained personality domains, suggesting that low‐dimensional structures may reflect phenotypic expressions of shared biological substrates [14, 15]. Moreover, converging cross‐cultural evidence has revealed robust consistency in such broad dimensions across linguistic and cultural contexts, supporting their potentially universal role in representing the latent architecture of human personality [10, 11].

However, instruments designed around the Big Five impose strong a priori constraints on trait covariance, typically prioritizing dimension‐level scores over finer‐grained item‐level structure. Converging evidence from large‐scale genomic studies reveals substantial genetic intercorrelations among Big Five dimensions and overlapping genetic associations with a range of psychiatric phenotypes [16, 17, 18], indicating that important covariance extends beyond individual trait dimensions. Additionally, personality questionnaire items themselves have been shown to contain reliable trait information beyond their shared variance with broader dimensions [19, 20]. Together, these observations suggest that alternative, cross‐cutting trait configurations may capture functionally relevant variance not fully represented in traditional Big Five summaries. This motivates the use of exploratory, data‐driven approaches that relax a priori constraints and derive covariance structure directly from item‐level data [19].

Recent methodological advances in machine learning, combined with the availability of large‐scale data resources [21, 22], provide promising opportunities to address these challenges. Specifically, these approaches enable the exploration of item‐level covariance within established personality inventories, to uncover alternative decompositions that may shed new light on aspects of personality structure that are not fully captured by classical models. Among these techniques, orthogonal projective non‐negative matrix factorization (OPNMF) offers a principled framework suitable for investigating questionnaire data [23]. By enforcing non‐negativity and additive composition, OPNMF enables complex trait patterns to be represented as parts‐based, transparent linear combinations of interpretable basis components [24]. Its projective nature further allows models learned on one sample to be directly applied to held‐out data, thereby enabling cross‐validated assessment of the stability and generalizability of these models across diverse populations [25]. Importantly, OPNMF's design balances mathematical constraints with psychological realism. While it imposes orthogonality and non‐negativity constraints on factor loadings to enhance interpretability, individuals’ factor scores remain unconstrained, preserving the natural correlational structure commonly observed in questionnaire data. This, in turn, allows systematic examination of inter‐factor relationships across different model resolutions.

These methodological advantages have previously produced useful insights across other psychological and clinical domains. In psychiatric research, for example, OPNMF has facilitated refined factorization of the Positive and Negative Syndrome Scale, contributing to the identification of schizophrenia subtypes and their neurobiological correlates [23]. In psychometrics, OPNMF was shown to produce a conceptually coherent and replicable decomposition of executive functioning that illustrated practical advantages over conventional factor‐analytic models [26]. Collectively, these findings underscore the capacity of OPNMF to balance interpretability and robustness when uncovering latent psychological structures [23, 26, 27].

Building on these developments, in the present study we investigated alternative, data‐driven decompositions of item‐level covariance within the Big Five personality questionnaires. Following the tradition of direct decomposition on large sets of personality items [10, 11], we applied OPNMF to a mega‐scale, globally diverse dataset containing responses to Big Five instruments, spanning wide‐ranging demographic and cultural backgrounds (*n* = 1,336,840; Table 1). By expressing personality as additive and part‐based item‐level covariation, this approach highlights recurring cross‐domain trait decompositions, complements traditional bipolar factor solutions, and supports direct out‐of‐sample projection. We then applied a state‐of‐the‐art analytical framework integrating OPNMF with cross‐validation to evaluate the stability and generalizability of trait decompositions. Furthermore, we examined the influence of demographic and cultural factors on trait organization, as well as the ability of these decompositions to explain variance across emotional/psychiatric, cognitive, and neural phenotypes.

**TABLE 1:** A mega‐scale dataset of personality ratings.

Dataset	Version	Item (*n*)	Participants (*n*)	Age	Countries / Regions (*n*)
IPIP‐NEO	IPIP‐120	120	619,150 (370,892 Female)	10‐99 (Avg = 25.19; SD = 10.22)	239
	IPIP‐300	300	328,306 (198,398 Female)	10‐99 (Avg = 25.25; SD = 10.05)	239
HCP‐D	NEO‐FFI	60	229 (125 Female)	16‐21 (Avg = 18.53; SD = 1.81)	1
HCP‐YA	NEO‐FFI	60	1,198 (650 Female)	22‐37 (Avg = 28.84; SD = 3.68)	1
HCP‐A	NEO‐FFI	60	725 (406 Female)	36‐100 (Avg = 59.89; SD = 15.75)	1
ESCS	NEO‐PI‐R	240	857 (375 Female)	18‐89 (Avg = 48.76; SD = 12.28)	1
BBC	BFI	44	386,375 (247,551 Female)	18‐130 (Avg = 35.97; SD = 13.86)	1

IPIP‐NEO: an IPIP‐based inventory designed to approximate the NEO‐PI‐R (Johnson, 2014); IPIP‐120/IPIP‐300 denote the 120‐item/300‐item versions used in the present response datasets; HCP‐D: Human Connectome Project Data of Development; HCP‐YA: Human Connectome Data of Young Adult; HCP‐A: Human Connectome Data of Aging; ESCS: Eugene‐Springfield Community Sample; BBC: British Broadcasting Corporation Personality Test Data

Beyond confirming the established Big Five structure, our analyses revealed an equally robust Big Two structure of personality traits. Both models exhibited high stability and generalizability, with only modest modulation by demographic and cultural variables. The Big Two framework captured distinct patterns of positive covariance among Big Five traits, distinguishing externally directed social engagement from internally directed affective–cognitive processing. Importantly, the spatial geometry of the Big Two landscape consistently aligned personality dimensions with key manifolds spanning the emotional/ psychiatric, cognitive, and neural spectrum. Together, these findings provide a theoretical and empirical basis for linking personality structure to the broader landscape of human psychological and biological phenotypes.

## 2 Results

### 2.1 Optimal Decomposition of Personality Traits Uncovers a Robust Big Two Structure

Our initial goal was to investigate the most optimal decomposition of personality traits using OPNMF. This method decomposes an original data matrix into two non‐negative matrices: one comprising factor loadings of psychological traits, and another representing individuals' scores on these factors. We defined an optimal solution as one that achieves both stability and generalizability in a comprehensive set of cross‐validation analyses (see Methods, Supplementary Methods, and Figures S1 and S2 for further details).

**FIGURE 1:**
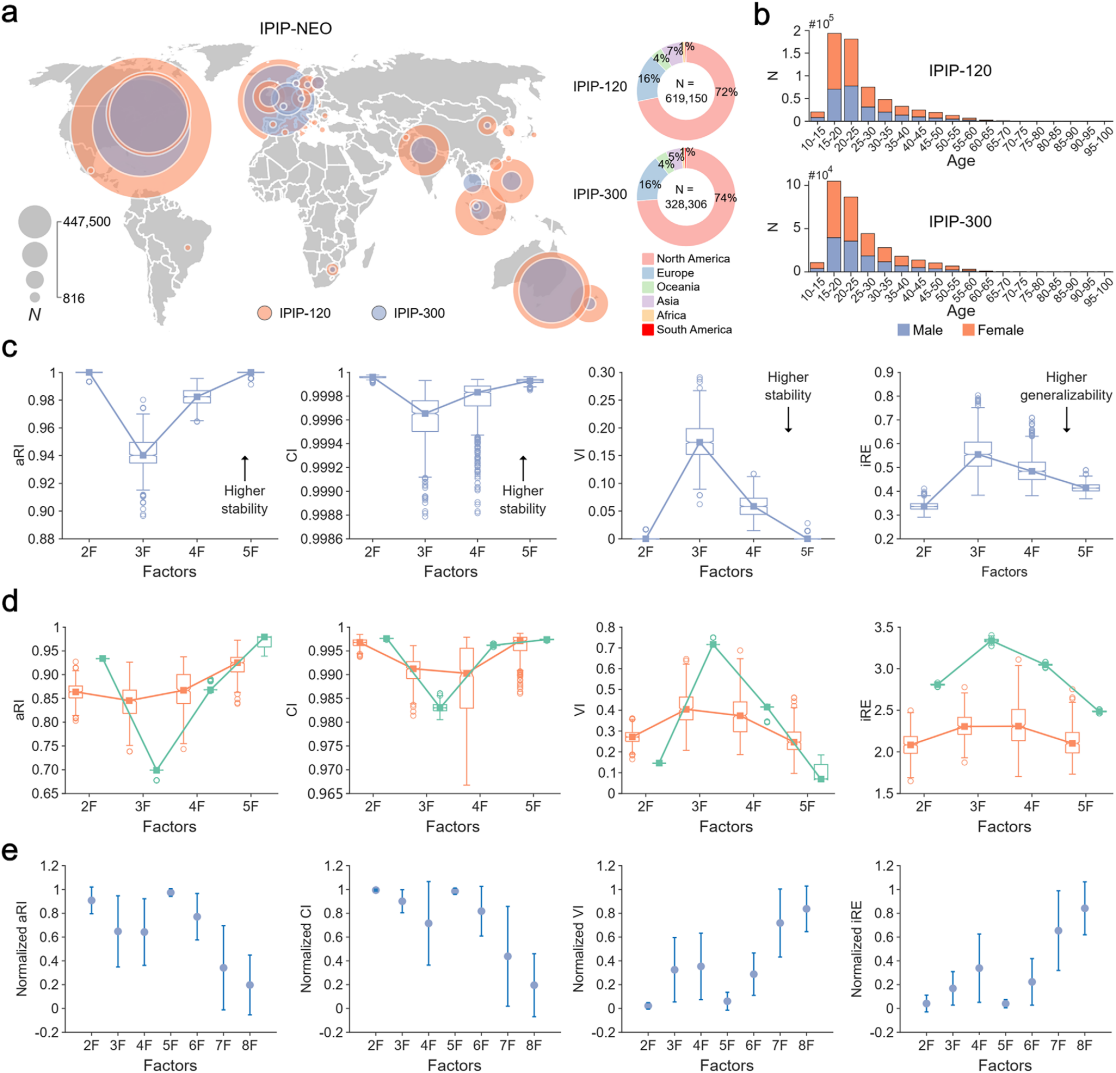
Optimal decomposition of personality traits. (a) Geographical distribution of the IPIP‐NEO responses, displaying sites with over 800 data points. (b) Distribution of sex and age in the IPIP‐NEO responses. (c) Model evaluation via cross‐validation. Evaluation indices, derived from 1,000 rounds of fivefold cross‐validation, demonstrate consistency of item assignments (higher aRI and lower VI) and similarity between basis matrices (higher CI and lower iRE) obtained from each iteration of the OPNMF‐based decomposition. Box plots illustrate the median, lower and upper quartiles, outliers (exceeding the 1.5 interquartile range), and non‐outlier maximum and minimum values. (d) Model evaluations incorporating culture (orange) and questionnaire versions (green). Stratified cross‐validation across countries/regions was used to examine the impact of cultural factors on the model, while bootstrap‐based cross‐validation between the IPIP‐120 and IPIP‐300 was employed to investigate the influence of questionnaire versions on the model. (e) Summary of model evaluations across a wider range of factor numbers (2‐8). Evaluation indices were normalized across assessments to facilitate comparisons. The mean values and 95% confidence intervals are displayed for each factor number.

For our main investigation, we used a mega‐scale, publicly available dataset containing responses to the IPIP‐NEO inventories (Johnson, 2014; IPIP‐ 120 and IPIP‐ 300; total *n* = 947,456), which collectively provides Big Five self‐report data from a geographically and demographically diverse international sample (Figure 1a,b; Table 1). We further complemented our analyses with additional samples from the Human Connectome Project (NEO‐FFI, *n* = 2,152), Eugene‐Springfield Community Sample (NEO‐PI‐R, *n* = 857) and the BBC personality survey (BFI, *n* = 386,375) to achieve a robust, stable and generalizable decomposition of personality traits within a mega‐scale composite dataset (*n* = 1,336,840). To help define the optimal trait decomposition, we quantified stability and generalizability using four complementary indices within a cross‐validation framework. Specifically, Adjusted Rand Index (aRI) and Variation of Information (VI) were used to assess the consistency of item assignments in trait decompositions, while Concordance Index (CI) was employed to evaluate the similarity, and Increased Reconstruction Error (iRE) was used to measure the generalizability of the model loading coefficients. Furthermore, we introduced an item‐level metric, termed Item Variability (IV), which facilitated the identification of questionnaire items with unstable latent factor assignments. Each index was estimated over 1,000 repetitions of fivefold cross‐validation, and performance was summarized using the median. We defined the optimal rank (i.e., number of factors) as the solution that jointly maximizes stability and generalizability, operationalized as higher aRI/CI and lower VI/iRE, with low IV.

At first, we applied OPNMF to the response data from the 120‐item questionnaire version of the IPIP, known as the IPIP‐120 (*n* = 619,150). Our evaluation across factors demonstrated notable stability and generalizability in both the two‐factor and the five‐factor models. In this extensive sample, where each of the five folds contained over 100,000 participants, we observed highly consistent factor affiliations and coefficient weights for both models, as indicated by close‐to‐ideal values across four key indices (Figure 1c). On the other hand, three‐ and four‐factor models demonstrated suboptimal performance.

With the aim of further evaluating the robustness of these results, we conducted a comprehensive set of analyses, examining the consistency of the models when accounting for common covariates such as culture or questionnaire versions (Figure 1d). We assessed cross‐cultural robustness using a cross‐validation stratified by country/region, ensuring that data from the same country/region did not appear in both training and test folds. When evaluating the potential influence of questionnaire versions, we employed a cross‐sample bootstrap method using the shared 120 items between IPIP‐120 and IPIP‐300. The four key indices across these assessments collectively reinforced the robustness of both the two‐factor and the five‐factor models, while three‐ and four‐factor models showed suboptimal performance (Figure 1d). Four additional assessments using only the IPIP‐300 inventory (Supplementary Methods and Table S1) further supported these findings, with results consistently favoring the two‐ and five‐factor models in the IPIP‐NEO (Figure 1e).

**FIGURE 2:**
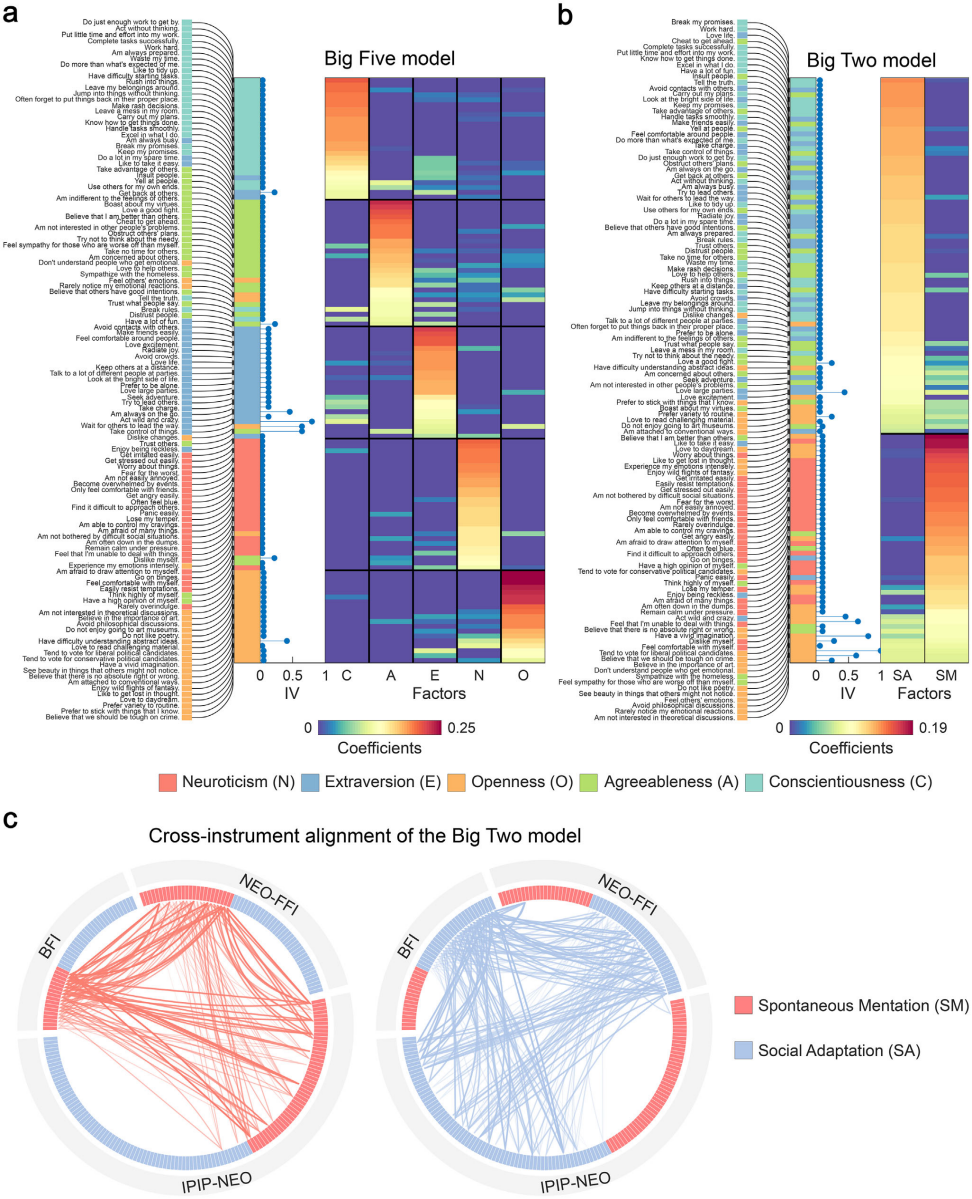
Big Two and Big Five models of personality. (a) OPNMF‐based five‐factor solution. (b) OPNMF‐based two‐factor solution. Items were assigned to the Big Five and Big Two models based on their maximal loading coefficients in the basis matrix generated from the full set of IPIP‐120 responses. Each factor is represented by a different color, corresponding to the theoretical factors/dimensions in the IPIP‐NEO, arranged vertically by their OPNMF basis matrix coefficients. Item Variability (IV) indicates the extent to which items may change their assignments during culture‐based cross‐validation. (c) Cross‐instrument semantic alignment of questionnaire items in the Big Two framework. Items from each instrument are ordered clockwise by the magnitude of their loading coefficients in their respective Big Two models. Connecting lines indicate pairs of items with the highest semantic similarity, and the thickness of the lines reflects the magnitude of the similarity values.

With regard to the factor structure, our OPNMF‐based five‐factor solution validated the widely accepted Big Five model. Most items aligned with their theoretical dimensions (Figure 2a), and the associations among those dimensions were preserved in the new decomposition (Figure S3). More significantly, the two‐factor solution revealed an alternative organizational principle that reconfigures items from different Big Five traits according to their shared positive variance patterns (Figure 2b). The first factor integrated items from Extraversion, Agreeableness, and Conscientiousness with relatively balanced contributions, representing externally oriented social functioning characterized by active engagement, cooperation, and goal‐directed self‐regulation. In contrast, the second factor showed a more concentrated weight distribution and was primarily defined by items from Neuroticism and Openness. Items with higher loadings specifically captured imagination‐driven thought (e.g., “Love to daydream,” and “Enjoy wild flights of fantasy”) and affective cognition (e.g., “Worry about things,” and “Get stressed out easily”). This pattern largely suggests that the factor reflects spontaneous mentation, in which affective and cognitive exploration shifts attention from external goals to internally directed imagination, rumination, and emotional reflection. Taken together, we refer to this newly identified structure as a Big Two model of personality, with the two factors labelled as Social Adaptation (SA) and Spontaneous Mentation (SM), respectively.

To assess the reproducibility of our findings across different instruments and samples, we applied our analytical framework to additional datasets: the NEO‐FFI from the HCP dataset (*n* = 2,152) and the BFI from the BBC personality survey (*n* = 386,375). Both two‐factor and five‐factor solutions emerged as optimal decompositions across these datasets (Figures S4 and S5; Table S2). The OPNMF‐based five‐factor solutions closely corresponded to the theoretical Big Five dimensions, replicating the patterns observed in IPIP‐120. Furthermore, the two‐factor solutions consistently grouped items from Extraversion, Agreeableness, and Conscientiousness into one dimension while clustering Neuroticism and Openness items into a second, capturing the positive covariance within each cluster (Figure S3). This structural consistency extended beyond dimensional architecture to the semantic content of individual items themselves. Sentence embedding analyses revealed that the Big Two derived from different questionnaire versions reflected highly similar personality descriptions (Figure 2c), demonstrating a robust and coherent psychological construct that transcends specific item formulations. Additionally, to verify that the identified factor structures, particularly the novel Big Two model, were not driven by acquiescence bias [28], we conducted a sensitivity analysis using within‐subject mean‐centered data and obtained solutions that were largely identical to the original results (Table S3).

OPNMF enforces non‐negativity, resulting in an additive, parts‐based factor decomposition that is distinct from traditional methods. To evaluate how these constraints shape the resulting structures, we compared OPNMF with principal component analysis (PCA) and exploratory factor analysis (EFA) across three representative personality inventories (IPIP‐120, NEO‐FFI, and BFI). The analyses revealed that OPNMF produced more stable and generalizable factor solutions than both PCA and EFA under cross‐validation (Figure S6), consistent with prior findings [26]. Although all three methods reproduced the theoretical Big Five dimensions (Figure S7), they diverged in their two‐factor solutions, with EFA showing the lowest stability (Figure S6). Moreover, because PCA (Figure S8) and EFA (Figure S9) allow for both positive and negative loadings, they typically result in bipolar dimensions that contrast opposing trait poles. In contrast, OPNMF's non‐negativity constraint precludes bipolarity, forcing the decomposition to identify unipolar factors that represent distinct, additive sources of variance. Nevertheless, the variance patterns captured by Social Adaptation and Spontaneous Mentation were not entirely absent in the traditional methods. When items in the PCA and EFA solutions were sorted by the sign of their loadings to distinguish the opposing poles (i.e., grouping positive against negative coefficients to inspect each pole independently), all approaches revealed a similar underlying structure. Specifically, positive covariance was consistently observed within the Neuroticism–Openness cluster and within the Extraversion‐Agreeableness‐Conscientiousness cluster, regardless of the employed method (Figures S8 and S9). Taken together, these findings indicate that the Big Two model reflects robust latent dimensions that persist across different measures, while OPNMF captures their variance in a particularly stable and parsimonious form.

Beyond model evaluation, our cross‐validation process also allowed us to assess the robustness of individual questionnaire items. While the main IPIP‐120 evaluation (Figure 1c) demonstrated minimal variability in item assignments, considering cultural influences highlighted a notable degree of item variability (Figure 1d). Further examination through culture‐based cross‐validation showed that while most items remained stable across two‐ and five‐factor models, items with lower coefficients showed higher variability (Figure 2a,b). For example, certain Openness items describing social interaction (e.g., “Feel others' emotions”) were assigned to Agreeableness. Conversely, certain Agreeableness items associated with high self‐esteem (e.g., “Think highly of myself”) were categorized under Neuroticism. Together, these findings highlight the potential influence of external factors on model stability and generalizability, forming the foundation of our subsequent analysis.

### 2.2 Optimal Personality Models are Applicable to Demographic and Cultural Subgroups

In addition to assessing the overall robustness of the Big Two and Big Five models, we further investigated their generalizability to specific subgroups and analyzed factors that may influence model generalization. Here, generalizability was quantified based on reconstruction error when applying the overall model to various demographic and cultural subgroups (see Methods). The demographic subgroups included two sex groups (male and female) and three age groups (teenagers, 10–17 years; adults, 18–59 years; senior adults, 60 years and older), while questionnaire responses from 31 countries/regions were categorized into cultural subgroups. We also investigated three additional factors that could potentially affect overall model generalizability: (1) sample size of the subgroup, (2) intra‐group heterogeneity, reflecting the magnitude of model fit error when the model is constructed exclusively from within subgroup data, and (3) similarity between the overall model and group‐specific models.

**FIGURE 3:**
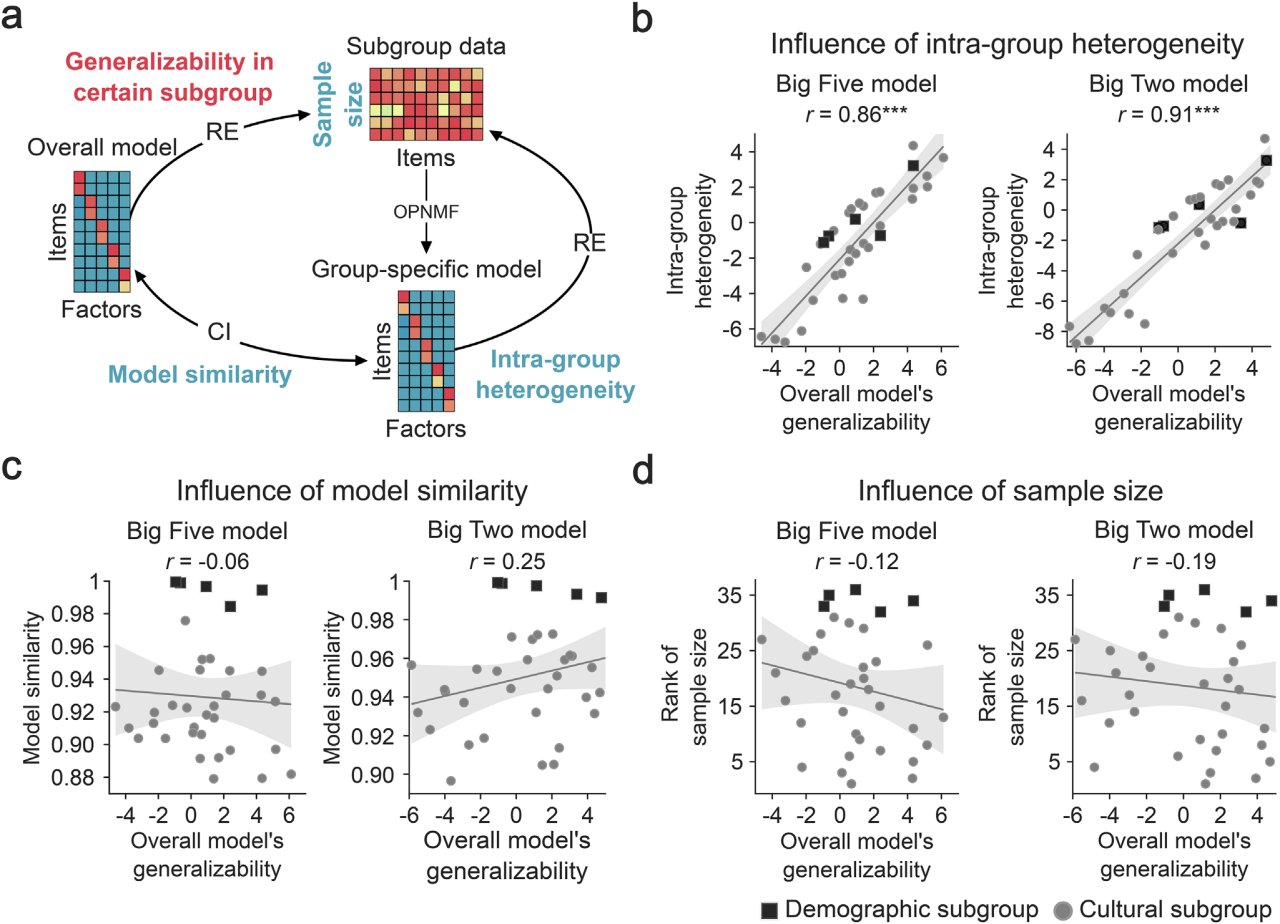
Factors influencing model generalizability. (a) The generalizability of the overall model across subgroups. The overall model generalizability was quantified by the reconstruction error (RE) when applying the overall model to demographic and cultural subgroups. The generalizability value was adjusted by null models derived from data with randomly assigned group labels (see Methods). Sample size indicates the number of questionnaire responses from each subgroup. The intra‐group heterogeneity was quantified through RE when applying population‐specific models to their respective subgroups (adjusted by null models). Model similarity was calculated using the concordance index (CI) between the overall model and group‐specific models. (b) Correlation between overall model generalizability and intra‐group heterogeneity. (c) Correlation between overall model generalizability and model similarity. (d) Correlation between overall model generalizability and subgroup sample size. *** *p* < 0.001, two‐tailed.

We found that the generalizability of the Big Two and Big Five models varied across different subgroups (Figure S10). The intra‐group heterogeneity consistently and independently accounted for over 70% of the overall model generalizability (Figure 3b; Big Five: *r^2^* = 0.74, *p* < 0.001; Big Two: *r^2^* = 0.83, *p* < 0.001, two‐tailed). Neither differences between the population‐specific models and the overall model (Figure 3c; Big Five: *r^2^* = 0.004, *p* = 0.72; Big Two traits: *r^2^* = 0.06, *p* = 0.14), nor the sample size of each subgroup (Figure 3d; Big Five: *r^2^* = 0.04, *p* = 0.25; Big Two: *r^2^* = 0.01, *p* = 0.48) could explain this variance. These results indicate that differences in model fit were primarily driven by intra‐group heterogeneity, which may stem from inconsistent factor structures or erratic questionnaire responses within the subgroups. Specifically, females exhibited lower intra‐group heterogeneity than males, adults and senior adults had lower intra‐group heterogeneity than teenagers, and intra‐group heterogeneity was relatively lower in most Asian countries/regions in comparison to the rest of the world (Figure S10).

While the extent of deviation across models based on age and sex was negligible (Figure 3c; Figure S11), culture‐specific models manifested moderately greater deviations (Figure 3c). Subsequent analyses indicated that culture‐specific Big Five models exhibited a non‐assortative community structure, with personality models in different countries/regions converging toward countries in the core Anglosphere. On the other hand, culture‐specific Big Two models displayed an assortative community structure, suggesting a systematic fine‐tuning within various geographically‐constrained cultural spheres (Figure S12). This fine‐tuning primarily adjusted the coefficients of theoretical dimensions within the two Big Two traits without changing the underlying factor structure, with the sole exception of Openness. In the Asian sphere, certain socially oriented Openness items contributed more strongly to Social Adaptation than to Spontaneous Mentation (Figure S13). Even so, these adjustments involved low‐weight items and exerted minimal influence on the final factor scores.

Taken together, these assessments indicate that the optimal Big Two and Big Five models generalize robustly across demographic subgroups. Variability in overall model generalizability is driven primarily by intra‐group heterogeneity rather than by model instability or subgroup sample size. In the cultural context, while the overall models exhibit broad generalizability across cultural subgroups, targeted fine‐tuning further enhanced model fit, particularly in the case of the Big Two model.

### 2.3 Geometry of the Big Two Model Space Reveals Manifolds of Neurocognitive Profiles

**FIGURE 4:**
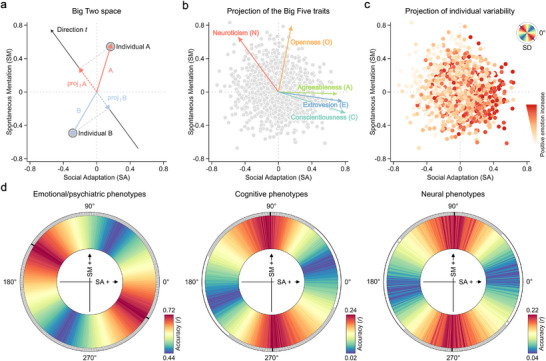
Manifolds of neurocognitive profiles captured by the Big Two space. (a) A 2D framework anchored by Spontaneous Mentation and Social Adaptation, where individual positions are uniquely defined by coordinate values along the two axes. This spatial representation naturally creates a polar coordinate system where vector orientation and magnitude reflect the balance and strength of factor expression. Through directional projections, this vector approach enables the estimation of various personality dimensions and phenotypic characteristics of individuals. (b) Directions in the Big Two space linked to the Big Five traits. Biplots illustrate the embedding of traditional Big Five traits within the Big Two space, while displaying individual Big Two factor scores in the HCP Young Adult dataset. Each Big Five trait appears as a vector whose orientation and magnitude reflect its Pearson correlations with both Big Two traits. (c) Big Two space as a framework for individual variability. Spontaneous Mentation and Social Adaptation exert a coordinated influence on phenotypic expression. For example, positive affect varies mainly along the principal diagonal, which captures the highest personality variance (measured by standard deviation [SD], shown with colors ascending chromatically from blue to red; black line denotes the peak direction). This pattern indicates that positive affect is not determined by either Big Two traits in isolation, but rather reflects their joint influence. (d) Prediction analysis within the Big Two space. The predictive models' use vector projections in specific directions as targets, while predictors span emotional/psychiatric, cognitive, and neural phenotypes. The analysis includes 180 predictive models, covering directional projections from 1° to 180° in the Big Two space. The inner ring shows mean predictive accuracy (correlation between actual and predicted projections) across 30 iterations of fivefold cross‐validation. The outer ring shows predictive significance from 5 000 permutation tests: gray denotes FDR *q* < 0.05 significance, while black marks peak accuracy position.

Having established the robustness of the Big Two model, we next explored how the newly identified personality traits can be explained by other aspects of human cognition and behavior. For that purpose, we established a two‐dimensional (2D) personality space anchored by the identified Social Adaptation and Spontaneous Mentation factors. In this space, each individual's personality appears as a unique vector with specific direction and strength, representing their Big Two patterns (Figure 4a).

As expected, this simplified 2D framework revealed robust associations between the Big Two and the Big Five traits, with Neuroticism and Openness aligning along one axis, while Extraversion, Agreeableness, and Conscientiousness align along the other (Figure 4b; Figure S14). Importantly, this condensed representational format enabled us to reliably estimate relationships with other features of human cognition and behavior. Through the analysis of directional vector projections, we uncovered systematic relationships between personality characteristics and distinct neurocognitive profiles (Figure 4c). Specifically, we investigated the neurocognitive relevance of personality traits within the Big Two space using thresholded partial least squares (T‐PLS) regression models [29]. These predictive models targeted vector projection lengths in specific directions within the Big Two space. The model features included a diverse set of phenotypic data across emotional/psychiatric symptoms, cognitive abilities, and brain functional connectivity profiles. By assessing the predictive accuracy of the model across various directions, we determined the strength of the association between different spatial directions and manifolds of neurocognitive profiles. For this analysis, we utilized NEO‐FFI data from the HCP Young Adult dataset, which offers extensive behavioral and neuroimaging assessments across 988 individuals.

T‐PLS regression analysis revealed that multivariate neurocognitive features reliably predicted trait characteristics corresponding to different orientations within the Big Two space (Figure 4d). Emotional/psychiatric features (*n* = 32) significantly correlated with personality traits across all directions, particularly with those near the primary diagonal (peak, 147°; *r* = 0.716 [0.715 0.718], *p* < 0.001, 5,000 random permutations). Cognitive features (*n* = 10) correlated with personality traits oriented from 51° to 156°, peaking around 90° (peak, 91°; *r* = 0.241 [0.238 0.243], *p* < 0.001). When applied to functional connectivity features of the brain (*n* = 419,070), the model significantly predicted personality traits oriented from 45° to 140°, with the strongest effects nearing 90° (peak, 89°; *r* = 0.223 [0.217 0.228], *p* < 0.001). These findings reveal significant links between different orientations within the Big Two space and manifolds comprising distinct combinations of phenotypic features. Notably, Spontaneous Mentation demonstrated more robust neural substrates and stronger cognitive associations compared to Social Adaptation, though both axes showed strong emotional/psychiatric correlates.

**FIGURE 5:**
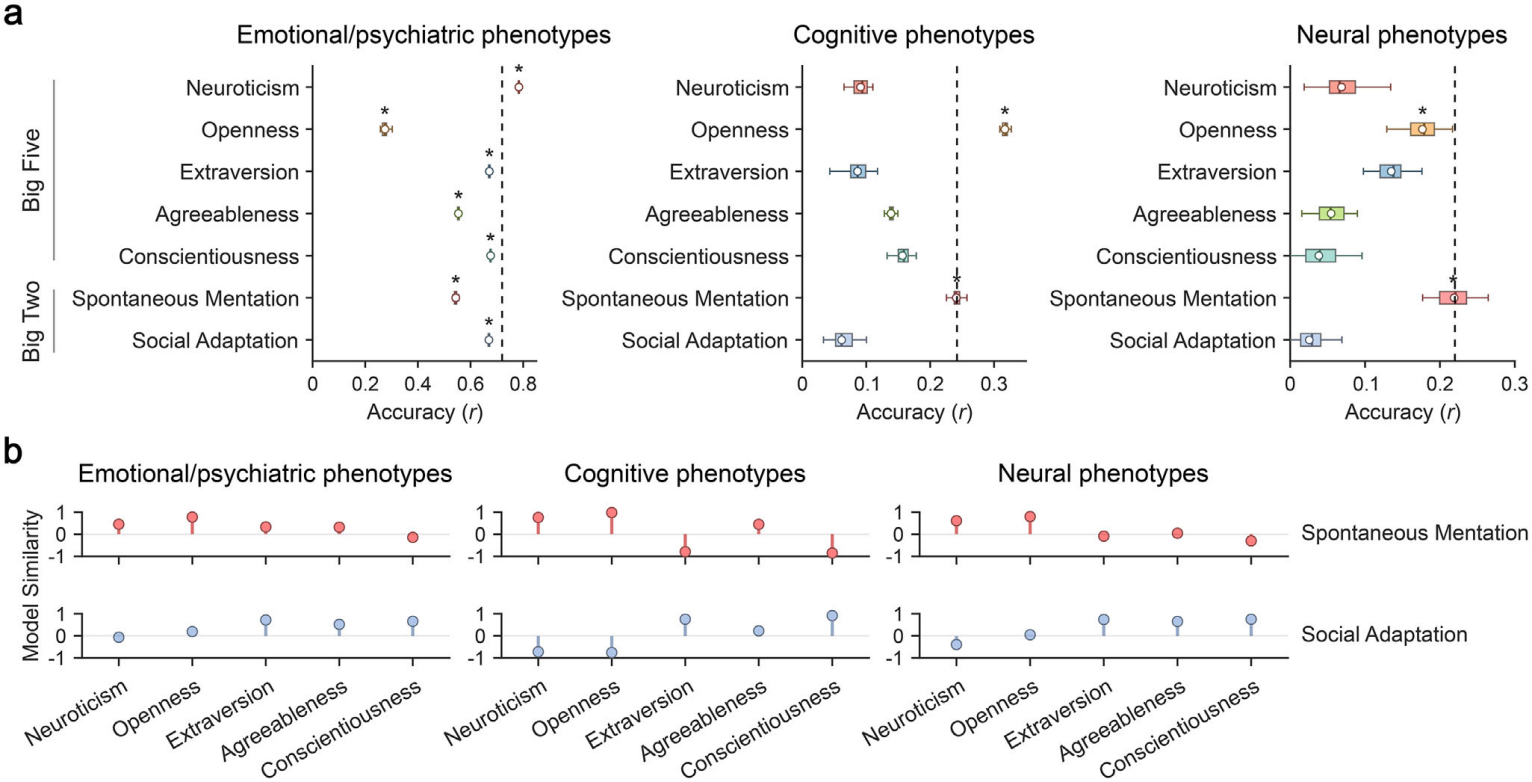
Comparison of predictive performance between the Big Two and Big Five models. (a) Prediction analysis across different domains. Boxes show the interquartile range of predictive accuracy across 30 iterations of fivefold cross‐validation, with lines representing the median and circles denoting the mean. The black dashed line represents the optimal mean prediction obtained within the Big Two space. * FDR *q* < 0.05. (b) Correlations of prediction model weights linking the Big Two and Big Five traits. A higher similarity in prediction model weights (Pearson correlation) indicates a closer phenotypic association pattern between traits.

To compare the predictive performance of the Big Two and Big Five frameworks, we applied T‐PLS regression analyses across different domains (Figure 5a). The Big Two space achieved the highest overall predictive performance and optimal accuracy for neural phenotypes. In contrast, the Big Five model showed domain‐specific strengths: emotional/psychiatric features most strongly predicted Neuroticism, while cognitive features best predicted Openness. Further analysis of prediction model weights revealed that the two Big Two axes aligned closely with distinct Big Five dimensions in their phenotypic association patterns (Figure 5b; Figure S15). Specifically, Spontaneous Mentation aligned with Neuroticism and Openness, whereas Social Adaptation aligned with Extraversion, Agreeableness, and Conscientiousness, confirming the integrative nature of the Big Two framework.

In summary, the Big Two framework links personality traits to neurocognitive profiles within a unified, 2D space. This two‐factor model captures the major positive covariance structure of Big Five items, improves prediction of neural phenotypes, and maintains systematic associations with emotional, psychiatric, and cognitive features. Its geometric properties provide a parsimonious coordinate system for mapping individual differences across multiple levels of analysis.

### 2.4 The Big Two Traits are Linked to Specific Emotional/Psychiatric, Cognitive, and Neural Phenotypes

After establishing the utility of a continuous Big Two space for capturing individual differences in personality, we examined its neurocognitive correlates by employing bootstrap testing to identify phenotypic features that robustly predict personality traits.

**FIGURE 6:**
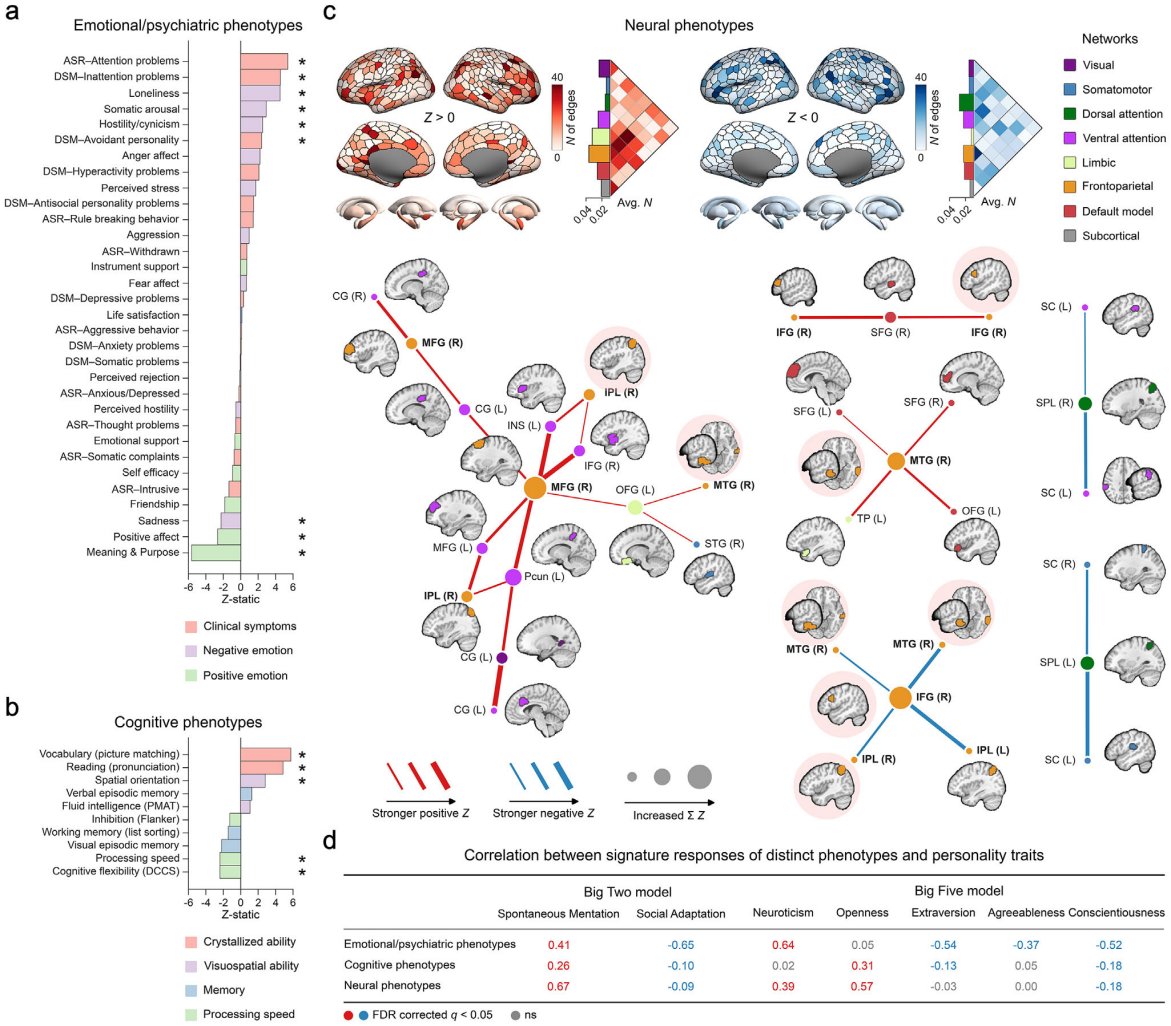
Behavioral and neural phenotypic associations in the Big Two space. (a) Predictive weights of the emotional/psychiatric model. (b) Predictive weights of the cognitive model. Statistical significance of predictive weights was determined using z‐statistics from a 5,000‐sample bootstrap procedure. * FDR, *q* < 0.05 (two‐tailed). (c) Top: Whole brain predictive weights of the neural model. Functional connectivity features (edges) are thresholded at uncorrected *p* < 0.05 (two‐tailed, bootstrap tests with replacements). Region weights (nodes) represent the cumulative count of significant edges. Edge counts are normalized at the network level by dividing by the number of edges that exist within or between networks. Bottom: Critical connections within the neural model. Edges are thresholded at FDR corrected *q* < 0.05. Brain regions showing both positive and negative edge weights are marked with a pink background. MFG, middle frontal gyrus; SFG, superior frontal gyrus; IFG, inferior frontal gyrus; IPL, inferior parietal lobule; MTG, middle temporal gyrus; CG, cingulate gyrus; INS, insular gyrus; Pcun, precuneus; OFG, orbitofrontal gyrus; TP, temporal pole; SPL, superior parietal lobule; SC, somatosensory cortex (d). Correlation between signature responses of different phenotypes (a–c) and personality traits. Signature responses are calculated using the dot product of the z‐statistics‐based model weight and the data. The chart values represent Pearson's correlation coefficients between signature responses and both Big Two traits and Big Five traits.

First, we examined the most predictive behavioral models within the Big Two space. For the emotional/psychiatric model, the most reliable model weights appeared along the 147° orientation. This orientation demonstrated strong and consistent associations with various psychiatric indicators and emotional characteristics (Figure 6a). Specifically, individuals with higher scores (i.e., the positive projection length along this direction) exhibited increased levels of psychiatric symptoms, particularly those related to attentional difficulties and avoidant behaviors, as well as heightened negative emotional experiences such as loneliness and somatic complaints. Conversely, individuals with lower scores in this orientation reported more positive emotional experiences, including a stronger sense of life meaning and purpose. These patterns are consistent with well‐established findings linking psychopathology to higher Neuroticism and lower Conscientiousness, Extraversion, and Agreeableness [30, 31]. Importantly, the Big Two framework provides a parsimonious integration of these relationships, with the 147° orientation capturing the joint influence of these personality dimensions on emotional regulation within a unified low‐dimensional space.

In the cognitive model, the 91° orientation exhibited the most reliable model weights, revealing systematic associations with various cognitive abilities (Figure 6b). Individuals scoring higher along this orientation demonstrated superior performance in measures of crystallized intelligence, such as vocabulary and reading, as well as enhanced visuospatial abilities, especially in spatial orientation tasks. However, these individuals performed comparatively worse in executive function tasks, particularly those measuring cognitive flexibility and processing speed. This orientation reflects the positive relationship between Neuroticism and Openness, indicating that their shared variation (i.e., Spontaneous Mentation) may differentially influence distinct components of cognitive functioning.

Brain functional connectivity architecture also exhibited a stable pattern (Figure 6c). Along the 89° orientation, which reflects the positive relationship between Neuroticism and Openness, distinct patterns emerged in both inter‐ and intra‐network connectivity. Enhanced connectivity appeared primarily in inter‐network connections involving the frontoparietal network (FPN), default mode network (DMN), limbic network (LN), and ventral attention network (VAN), with FPN emerging as the main contributor. Conversely, reduced connectivity was most evident within the FPN's internal connections, and in the inter‐network connections between the dorsal attention network (DAN) and FPN, as well as connections involving the VAN. At the regional level, right FPN emerged as a critical hub: connections linking the right posterior middle temporal gyrus, inferior frontal gyrus, and inferior parietal lobule showed consistently reduced connectivity strength. However, these same regions demonstrated enhanced connectivity when interacting with the cingulate gyrus, medial frontal areas, and anterior temporal regions.

Moreover, signature responses, which represent composite feature scores derived from model weight combinations, exhibit significant yet distinct associations with both the Big Two and Big Five personality frameworks (Figure 6d). The Big Two demonstrated superior performance in capturing neural phenotypes, emphasizing generality across phenotypic domains, whereas the Big Five revealed trait‐specific associations through distinct phenotype mappings.

Collectively, the Big Two space captured shared variance in emotional/psychiatric symptoms and cognitive abilities, specifically within crystallized, visuospatial, and executive control functions. Furthermore, FPN connectivity with other association networks emerged as the primary neural substrate underlying this space (Figure S16).

## 3 Discussion

The main objective of our study was to assess the most optimal and alternative decomposition of human personality using advanced machine learning methods and a mega‐scale global dataset. First, through a series of model evaluation procedures, our initial results validated the robustness of the classical Big Five model across multiple datasets. Importantly, we identified a novel Big Two model comprising Spontaneous Mentation and Social Adaptation, which capture positive covariation within a Neuroticism‐Openness item cluster and an Extraversion‐Agreeableness‐Conscientiousness item cluster, respectively. Both the Big Five and the newly identified Big Two models showed minimal influence from age, sex, or culture, with model fitting errors attributable primarily to within‐sample heterogeneity rather than intrinsic differences between personality frameworks or sample size variations. Furthermore, the construction of a 2D Big Two space provided a parsimonious framework capturing robust associations across cognitive, mental health, and neural phenotypes. This lower‐dimensional representation achieved superior predictive performance for brain functional connectivity compared to the Big Five, while maintaining comparable associations with behavioral domains. We propose that the Big Two model may serve as a neurocognitively grounded scaffold for integrating personality research across interdisciplinary domains.

Our study demonstrates the value of applying advanced machine learning methods, such as OPNMF, to personality research. The OPNMF‐based decomposition shares methodological commonalities with exploratory factor analysis applied to large sets of personality descriptors [10, 11], in that both approaches perform dimensionality reduction on item covariance structures to recover latent factors that organize observed personality descriptors. However, unlike traditional methods that produce global, bipolar components with overlapping item contributions, OPNMF enforces non‐negativity and orthogonality constraints that enable purely additive decomposition, producing interpretable, localized patterns with distinct item‐to‐factor assignments [32, 33]. This approach not only replicates the Big Five model but also uncovers a novel Big Two structure with enhanced stability and generalizability compared to conventional factor‐analytic methods, aligning with recent applications demonstrating OPNMF's potential for psychometric data analysis [23, 26, 27].

Applying the OPNMF framework, we first validated the Big Five model through rigorous cross‐validation. The five‐factor solution exhibited robust stability and generalizability across samples, questionnaire versions, cultural contexts, and demographic subgroups, refuting skepticism about its cross‐population applicability and confirming its status as a stable structure of human personality [34]. However, not all items demonstrated equivalent representativeness within their assigned dimensions. Low‐coefficient items showed deviation from theoretical assignments, particularly in the IPIP‐NEO responses, compared to the NEO‐FFI and BFI responses where item assignments aligned more closely with theory. These findings underscore the value of item variability indices for assessing stability in questionnaire design [35] and highlight the importance of sparsity constraints in emphasizing substantive contributors during factor decomposition [23].

Of greater significance, our analysis revealed a novel two‐factor decomposition that highlights previously underappreciated patterns of covariance among items in Big Five questionnaires. One factor emerged from a balanced integration of Extraversion, Agreeableness, and Conscientiousness items, characterizing externally oriented social functioning marked by engagement, cooperation, and goal‐directed regulation, traits consistently implicated in collaborative problem‐solving and prosocial decision‐making [36, 37]. The other factor showed concentrated loadings from Neuroticism and Openness items capturing imagination‐driven thought and affectively charged cognition, reflecting internally directed mental activity in which attention shifts from external goals toward spontaneous imaginative exploration, emotional processing, and self‐generated thought [38, 39]. Given these distinct orientations, we termed these factors as Social Adaptation and Spontaneous Mentation, which together constitute a novel Big Two model.

While previous research has documented shared variance among Extraversion, Agreeableness, and Conscientiousness [40], Social Adaptation highlights their deeper functional unity as an integrated social‐regulatory system. Extraversion and Agreeableness facilitate positive interactions and relationship quality, whereas Conscientiousness elicits social recognition through reliable, competent behavior that fosters reciprocal obligations [1, 41]. Together, these traits enhance coordination and social efficacy in cooperative contexts [36] and promote prosocial motivation through sensitivity to social rewards and affiliative goals [37]. This pattern conceptually parallels the Communion/Social Self‑Regulation dimension identified in cross‐cultural two‐factor models of personality [10, 11], which emphasizes other‐oriented functioning and social norm adherence. However, a key distinction emerges in factor composition: whereas Communion/Social Self‐Regulation distributes across warmth‐morality aspects (primarily Agreeableness) and self‐control aspects (primarily Conscientiousness), Social Adaptation integrates Extraversion, Agreeableness, and Conscientiousness more evenly, suggesting a unified dimension of socially oriented functioning that encompasses engagement, cooperation, and goal‐directed behavior within a single coherent construct. Moreover, its negative associations with negative emotionality and psychiatric symptoms further support its protective role as a psychological resource that generates supportive relationships and buffers distress. We therefore conceptualize Social Adaptation as reflecting the motivation and capacity for constructive engagement in social life, through which individuals cultivate supportive interpersonal resources that promote resilience and sustain psychological well‐being.

In contrast to Social Adaptation's outward orientation, Spontaneous Mentation reflects an inwardly focused mode of functioning defined by the convergence of affective volatility and memory‐based cognition. This dimension primarily integrates Neuroticism with select Openness aspects capturing introspective and imaginative engagement, diverging conceptually from the Agency/Dynamism dimension in classic two‐factor models [10, 11]. Whereas Agency/Dynamism combines surgency and social engagement (primarily Extraversion) with intellectual curiosity (aspects of Openness) to represent outward‐directed, goal‐driven agency, Spontaneous Mentation links emotional reactivity with memory‐based processing to characterize internally oriented mental activity operating independently of instrumental social goals. This distinctive configuration illuminates previously obscured connections among personality structure, cognitive functioning, and psychopathology. Genetic evidence indicates that Neuroticism and Openness share genetic variance with psychiatric conditions such as schizophrenia and bipolar disorder [16, 17, 42, 43, 44], suggesting that Spontaneous Mentation captures a latent susceptibility dimension wherein affective lability converges with imaginative processing. While Openness is firmly established as the primary Big Five correlate of intelligence, Neuroticism typically exhibits negative or negligible associations with general cognitive ability [45]. Our results refine this understanding by isolating the specific variance shared between these traits. We found that Spontaneous Mentation is associated with enhanced crystallized and visuospatial abilities yet reduced executive control functions. These patterns align with mind‐wandering and spontaneous thought frameworks [38, 39], wherein internally directed mental activity facilitates knowledge acquisition and creative elaboration while increasing vulnerability to emotional dysregulation and goal‐directed attentional regulation.

Concurrent with its behavioral profile, Spontaneous Mentation shows distinctive frontoparietal network signatures. Higher levels were linked to greater frontoparietal integration with the default mode, limbic, and ventral attention networks, alongside weaker coupling with the dorsal attention network and reduced within‐network connectivity. This pattern suggests a trait‐level redistribution of frontoparietal coupling across networks supporting externally versus internally oriented cognition. The dorsal attention network is centrally implicated in allocating attention to perceptual input [46], whereas the default mode network supports internally generated thought, including autobiographical and self‐referential mentation [47]. Accordingly, greater frontoparietal connectivity with the default mode network together with reduced connectivity with the dorsal attention network may index an individual‐difference shift toward internally generated processing. Such a shift could support knowledge‐based cognition while also increasing vulnerability to diminished goal maintenance and executive control, consistent with the opposing behavioral associations observed in this study. In line with this interpretation, increased frontoparietal to default mode connectivity has been associated with broad psychopathology liability [48] and poorer executive control [49], while related cross‐network integration has also been implicated in creative cognition [50]. Collectively, these findings suggest that Spontaneous Mentation captures a coordinated brain‐behavior axis in which frontoparietal connectivity profiles reflect a trade‐off between internally oriented cognitive strengths and regulatory vulnerability within normative populations.

Building upon these findings, we propose that the Big Two space delineated by Social Adaptation and Spontaneous Mentation offers a parsimonious coordinate system for organizing individual differences in personality. Within this framework, the largest personality variations emerge along the diagonal, demonstrating that both traits jointly contribute to shaping individual personality profiles. While projecting variables into low‐dimensional personality space is well‐established practice, our contribution lies in identifying a novel two‐factor structure that meaningfully reorganizes Big Five content according to functional orientation, specifically distinguishing externally directed social engagement from internally directed mental activity, and demonstrating its coherence with neurocognitive patterns. Beyond its descriptive parsimony, this reformulation carries both theoretical and practical implications. Theoretically, it aligns with emerging frameworks that conceptualize psychological experience as trajectories through low‐dimensional state spaces shaped by attractor dynamics [51]. Within this perspective, the Big Two dimensions may represent attractor basins that channel how experience unfolds and stabilizes: individuals high in Social Adaptation gravitate toward externally scaffolded routines, whereas those high in Spontaneous Mentation are drawn toward internally generated exploration. This framework may also have practical value. For example, Spontaneous Mentation enables identification of individuals who combine cognitive strengths with emotional vulnerabilities, informing interventions that leverage intellectual capacities while addressing regulatory difficulties. Thus, the Big Two model offers a parsimonious lens for understanding both the structural organization of personality traits and the dynamic processes governing experiential adaptation.

Moreover, our results affirmed the soundness of constructing a unified model, as the fitting errors in model application primarily emerged from intra‐group heterogeneity across various demographic and cultural subgroups. Still, we observed subtle culture‐specific tuning, most apparent within Openness. Prior work has emphasized cross‐cultural mean differences in Big Five trait levels, with Western samples often showing higher mean Openness ratings [52, 53]. Our findings provide a complementary view, suggesting that part of the variation arises from fine‐grained reweighting of items rather than mean shifts alone. A plausible interpretation is that facets of Openness are expressed in a culturally congruent manner. For instance, curiosity may be framed to prioritize social harmony, or aesthetic preferences may be shaped by collective norms. Such expressions could align more closely with affiliative self‐regulation in East Asian cultural contexts [54, 55, 56]. Consequently, analyses based solely on average scores may understate culturally patterned dispersion within a trait. Because these adjustments concentrate on low‐weight items, a unified, sparsity‐based model still captures the dominant, cross‐culturally stable variance while accommodating modest cultural tuning.

It is crucial to distinguish the methodological nature of the Big Two structure identified here from established higher‐order factor models, such as Stability and Plasticity derived from Big Five intercorrelations [8, 9] or the lexical Big Two derived from adjective‐based descriptor sets [10, 11]. Traditional factor analytic methods like EFA and PCA allow for both positive and negative loadings, and are often obliquely rotated in psychometric practice to produce correlated dimensions. As a result, they tend to emphasize bipolar dimensions defined by opposing poles. For example, Stability is frequently expressed as higher Agreeableness and Conscientiousness combined with lower Neuroticism. In contrast, OPNMF minimizes reconstruction error under non‐negativity and orthogonality constraints on the loading vectors, resulting in sparse, additive, parts‐based components [32, 57]. By strictly enforcing orthogonality in the loading space, OPNMF inherently prevents oblique factor rotations. This modeling choice fundamentally shapes the recovered factor patterns by prioritizing the representation of personality as a sum of constituent parts. Accordingly, our solution can be best interpreted as a decomposition of the item‐level covariance structure into discrete, non‐overlapping building blocks. This parts‐based representation produces additive clusters that cut across the Big Five domains, rather than yielding bipolar axes that are typically derived from domain‐score correlations.

The non‐negativity constraint nevertheless warrants consideration in psychological settings where indicators may reflect opposite poles of a single underlying continuum. When item polarity is ambiguous, a bipolar construct could in principle be partitioned into two components capturing opposite poles. However, OPNMF imposes orthogonality on loading patterns rather than on participants’ factor scores, so the scores are free to correlate. This property offers a straightforward diagnostic. If two components mainly reflect opposite ends of a single continuum, their factor scores should be very strongly negatively correlated across participants, indicating functional redundancy rather than distinct traits. In the present study, the two components did not exhibit such redundancy, supporting their interpretation as separable dimensions of item co‐variation rather than an artifact of the sign constraints. More generally, we recommend that applications of OPNMF to less structured item sets routinely report factor score correlations and benchmark results against signed, potentially oblique factor models to verify that the recovered structure is not a sign‐induced partition of a single bipolar trait.

The current study has several important limitations. First and most critically, our findings are constrained by reliance on Big Five inventories, meaning the extracted Big Two factors reflect covariance patterns within that specific framework rather than representing a universal personality structure. Whether Social Adaptation and Spontaneous Mentation generalize beyond Big Five measures remains an open question requiring investigation with broader, less theory‐bound personality descriptors. Moreover, while our sensitivity analyses accounted for acquiescence bias, the influence of other response styles, such as socially desirable responding, cannot be ruled out without multi‐informant data. Second, although we demonstrated theoretical coherence with neurocognitive patterns, the range of external phenotypes examined was limited. More comprehensive characterization of this personality landscape requires extending investigations to more extensive behavioral, cognitive, and neural domains in larger and more diverse populations. Third, while our emphasis on low‐dimensional structure provides a parsimonious organizing framework, item‐level analyses reveal meaningful heterogeneity meriting further investigation. For instance, Openness items capturing introspective and imaginative thought covaried positively with Neuroticism on Spontaneous Mentation, whereas some items with low coefficients aligned more closely with sociability‐related Social Adaptation in IPIP‐NEO. This item‐level variability, particularly pronounced across cultures, suggests distinct sub‐factors within broader trait domains. Fine‐grained facet‐level analyses in diverse samples are needed to clarify these structural nuances and delineate the boundaries of the proposed framework.

Overall, using big data and machine learning methods, we revealed a novel two‐factor model of personality. The Big Two space defined by Social Adaptation and Spontaneous Mentation not only reveals previously underappreciated covariance patterns among Big Five items, but also provides a coherent organizing framework for mapping individual differences across neurocognitive profiles. This parsimonious landscape may thus provide substantial prospects for the theoretical integration of personality traits into more refined investigation of human cognition and behavior.

## 4 Methods

### 4.1 Personality Datasets

This study is based on publicly available datasets, the acquisition and release of which were all approved by the local reviewing boards of participating institutions and curated in accordance with the approved protocols. The reuse and analysis of this data was approved by the local Institutional Review Board, adhering to the data use agreements of each dataset and the relevant rules and regulations outlined in the Declaration of Helsinki for experiments involving humans.

To achieve a highly robust decomposition of personality traits that considers factors such as variations across samples, questionnaire formats, and demographic variables, the current study incorporated multiple large‐scale datasets. All the personality questionnaires employed in our study are rooted in the Big Five model (FFM) of personality, albeit in distinct and varying forms. Participants were asked to evaluate their agreement with various personality trait statements using five‐point Likert scales across all questionnaires. Negatively keyed items were reverse‐scored according to each instrument's standard scoring key prior to missing‐value handling and all subsequent analyses, such that higher values consistently indicated higher standing on the intended trait pole. The curated mega‐scale personality dataset includes:

#### 4.1.1 International Personality Item Pool Representation of the NEO PI‐R (IPIP‐NEO)

The International Personality Item Pool (IPIP) is a public‐domain repository of personality items and scales (https://ipip.ori.org). Here, we analyzed publicly available response data from web‐administered Big Five inventories constructed from IPIP items, specifically the IPIP‐120 and IPIP‐300 [58]. The IPIP‐300 examines domains similar to those in the Revised NEO Personality Inventory (NEO PI‐R) [2], but with an extended assessment of the five‐factor model. The IPIP‐120 employs a condensed version with a subset of 120 items, the psychometric properties of which have been validated to compare favourably to the IPIP‐300 [58]. In addition to five broad traits, both questionnaire versions assess 30 facets or subdomains of personality. The current study included independently collected responses from participants who filled out the web‐based questionnaires of IPIP‐300 (*n* = 328,306) and IPIP‐120 (*n* = 619,150). Johnson's validity criteria [59] were utilized to eliminate duplicate entries, inattentive or largely missing responses (larger than 10 responses), or insufficient internal consistency.

#### 4.1.2 NEO Five‐Factor Inventory (NEO‐FFI) and Revised NEO Personality Inventory (NEO PI‐R)

The NEO‐FFI [60] and NEO‐PI‐R [61] consist of 60 and 240 items, respectively. The NEO‐PI‐R was designed to assess the Big Five traits and its subdimensions. The NEO‐FFI items were extracted from the NEO‐PI‐R items that showed the strongest relationships with their respective domain factor scores, regardless of the item's intended facet. Hence, the 30 NEO‐PI‐R facets are not equally represented by the NEO‐FFI items.

In the current study, the NEO‐PI‐R data was obtained from the Eugene‐Springfield Community Sample with 857 participants [62], while the NEO‐FFI data was provided by the HCP dataset [63]. Since the NEO‐FFI is considered more user‐friendly, brief, and less time‐consuming than the NEO‐PI‐R, it has been regarded as one of the most widely accepted measures of the Big Five model [60]. Consequently, our assessment predominantly relied on the NEO‐FFI. Here, we included 2,152 NEO‐FFI responses from three publicly available projects [64], including HCP Young Adult (HCP‐YA), Aging (HCP‐A), and Development (HCP‐D).

#### 4.1.3 Big Five Inventory (BFI) from the British Broadcasting Corporation (BBC) Survey

The BBC personality survey [65] was advertised and promoted through various BBC media channels. The survey employed the 44‐item BFI [66], which assesses the Big Five personality dimensions using brief descriptive phrases rated on a 5‐point Likert scale. While the BFI shares the same theoretical framework as the NEO and IPIP inventories, it is more concise and employs independently developed items with distinct content and phrasing to measure the Big Five dimensions. The primary objective of the BBC dataset was to map personality distribution across the United Kingdom. Accordingly, only participants who reported living in England, Wales, or Scotland and completed the full personality questionnaire were included, resulting in a final sample of 386,375 responses.

### 4.2 Behavioral and Neural Phenotypes in the HCP‐YA Dataset

The HCP‐YA dataset was employed to investigate whether personality traits are reflective of distinct neurocognitive profiles among participants. Behavioral data were sourced from the NIH Toolbox behavioral measures as well as the Achenbach Adult Self‐Report (ASR) Syndrome Scales and the ASR DSM‐Oriented Scales [67]. Our analyses focused on two pivotal behavioral dimensions: cognition (*n* = 10) and emotional/psychiatric symptoms (*n* = 32). With regard to neural phenotypes, we directed our analysis toward functional connectivity data quantified based on resting‐state functional magnetic resonance imaging (fMRI) [68]. Phenotypic association analyses have demonstrated that resting‐state functional connectivity, as opposed to structural measures, often yields higher effect sizes and increased robustness [69].

### 4.3 Resting‐State Functional MRI Data Processing

The resting‐state functional MRI data was downloaded in its minimally preprocessed form, that is, after motion correction, B0 distortion correction, co‐registration to T1‐weighted images and normalization to Montreal Neurological Institute (MNI) space. Detailed descriptions of the minimal preprocessing pipeline are available in the original publication [70]. Following minimal pre‐processing, non‐smoothed functional imaging data were denoised using the anatomical component‐based correction (aCompCor) method [71, 72], which included noise components from cerebral white matter and cerebrospinal areas, and movement parameters (6 motion parameters, 6 temporal derivatives, and their squares) [73, 74]. To further reduce the impact of head motion, time points with FD >0.5 mm as well as the two succeeding and one preceding volumes, were regarded as regressors to reduce the spillover effect of head motion by the scrubbing method [75]. Finally, linear trends in time courses were removed, and ‐low‐frequency drifts and high‐frequency noise were removed using bandpass temporal filtering (0.008, 0.09 Hz).

After preprocessing, fMRI time series were parcellated into 416 regions of interest (ROIs) comprising 400 cortical [76] and 16 subcortical areas (excluding the cerebellum and brainstem) as defined in Freesurfer [77]. The extraction of time series data was accomplished by computing the mean of the voxel values contained within each parcel. Following this, a 416 × 416 matrix was created by calculating pairwise Pearson's correlation of the parcel time series. This process was applied to uncensored frames from each functional MRI run. The correlation matrices were then subjected to Fisher's z‐transformation, averaged across different runs, and transformed back into correlation values. Only participants (*n* = 988) who had completed all resting‐state fMRI scans were included in the final analysis.

### 4.4 OPNMF‐Based Factor Decomposition of Personality Traits

We employed OPNMF [32, 57] to extract latent factors from Big Five questionnaire data. Unlike traditional methods such as principal axis factoring or principal component analysis, OPNMF imposes a non‐negativity constraint, ensuring that all components contribute additively to observed patterns. This approach facilitates the identification of interpretable, sparse, and parts‐based representations, aligning naturally with the additive and hierarchical structure of personality traits (see Supplementary Methods for details). OPNMF has proven to be a robust, computationally efficient, and generalizable tool for psychometric research [23, 26, 27].

Specifically, OPNMF uses orthonormality constraints to promote orthogonal, sparse, and highly interpretable factor decomposition [23]. The decomposition generates two non‐negative matrices (Supplementary Methods and Figure S1): (1) a factor loading matrix W (or basis matrix) with factors as columns that can be easily generalized to new data due to projective constraint and (2) a factor‐score matrix *H* representing the summarized scores of individual participants along these factors. Importantly, OPNMF enforces orthogonality among factor loading vectors to identify distinct, non‐redundant patterns of item covariation, while leaving participants’ factor scores unconstrained and therefore free to correlate naturally in the data. The resulting sparse representation provides a clustering‐like structure that facilitates item assignment to specific factors while retaining assignment weights.

### 4.5 Main Evaluation Framework for Factor Decomposition

Using a variety of cross‐validation pipelines, the optimal number of factors ranging from 2 to 8 was investigated across personality datasets. The general framework (Figure S2) is as follows: (i) The personality trait decomposition was evaluated based on the behavioral questionnaire data containing *N* (number of observations) × *M* (number of items) matrix. The category labels (e.g., questionnaire version, data sources, kinship and demographic category variables) were assigned for which the evaluation considered the influence of data categories though different perspectives. (ii) Handling missing values by interpolation method. (iii) Cross‐validation through K‐folds or bootstrap methods, in which, the data was always assigned into the main sample and hold‐sample several times. The cross‐validation was performed 1,000 times across all evaluations. (iv) Applying OPNMF to generate basis matrices for both samples, and separately assigning items into different factors according to the maximal loadings in these basis matrices. (v) Evaluating different factor models (2‐8 factors) based on a set of evaluation indices. To mitigate the impact of outliers, we employed the median value of each evaluation index across 1,000 iterations as the basis for model assessment.

Here, different cross‐validation strategies were employed to determine the optimal factor decomposition, with the principal evaluation conducted using fivefold cross‐validation (5F‐CV). Additionally, bootstrap method was utilized in conducting cross‐sample or cross‐version evaluations. The choice of cross‐validation procedure was based on the evaluation of interest and data characteristics (see Supplementary Methods for details). For example, to examine the stability and generalizability of the models across cultures for IPIP‐120, we applied culture‐based 5F‐CV, that respected the culture effects by never splitting data in the same countries/regions between folds. Additionally, to assess the possible impact of item versions, we employed the cross‐sample bootstrap method on the shared items in both IPIP‐120 and IPIP‐300, as well as between NEO‐FFI and NEO‐PI‐R.

### 4.6 Model Evaluation Indices

We assessed the quality of the personality models using four distinct indices (see Supplementary Methods for details). Briefly, the adjusted rand index (aRI) measures the similarity between two sets of item assignments by considering both the number of pairs in agreement and the chance level of agreement, providing a normalized score that reflects the true level of clustering correspondence beyond random chance [78]. The variation of information (VI) metric serves to quantify the shared information between two item assignments [79]. It measures the quantity of information that is either lost or gained in the transition from one item assignment to another. Apart from the hard‐assigned items, OPNMF generates the basis matrix W, which allows an item to contribute to multiple factors or dimensions. Hence, the concordance index (CI) was employed to assess loading‐pattern stability by comparing the item–item cosine‐similarity matrices derived from the basis matrices [80]. The increased reconstruction error (iRE) quantifies out‐of‐sample generalizability as the increase in reconstruction error when applying a basis learned in one sample to reconstruct held‐out data, relative to within‐sample reconstruction [23]. To further detect the frailties of items during cross‐validation, an item level index of variability was also applied. The item variability (IV) index quantifies the consistency of an item's factor affiliation across different item assignments by calculating the relative overlap between the item sets associated with that factor across assignments [81].

### 4.7 Cross‐Instrument Validation of Factor Structure Consistency

We assessed the consistency of factor structures across questionnaires using three complementary criteria. First, we examined whether each factor consistently drew on the same sets of items from the theoretical Big Five dimensions across instruments. Second, for the two‐factor solutions, we quantified semantic similarity between items using sentence embedding techniques (see Supplementary Methods) to test whether items loading on the same factor showed greater semantic coherence across questionnaires than items loading on different factors. Third, we evaluated whether the correlation structure among factor scores was stable across datasets and instruments, focusing in particular on the pattern of correlations between the two‐factor and five‐factor solutions.

### 4.8 Assessment of Model Generalizability to Various Subgroups

Beyond assessing model quality through cross‐validation, we investigated error sources associated with the application of the overall model, developed using the complete sample, to various subgroups. To achieve this, we employed the overall model derived from the entire IPIP‐120 sample and assessed its generalization performance on subgroups with different sex, age and county/region labels, computing reconstruction error as a metric for assessing model performance. The lower the reconstruction error is, the better the model can be applied to explain the variation in a specific subgroup. Subsequently, a one‐sample t‐test was executed to compare the reconstruction error resulted from the actual labels with those generated from 100 randomized labels. During this process, demographic or cultural labels were randomized in the data, while the sample size under each label remained unchanged. Here, the model generalizability was identified by the average “actual‐random” differences of reconstruction error when using the overall model. If the “actual‐random” differences are significantly lower than zero, the overall model can be considered to better explain the variation in a given subgroup.

Next, we investigated which factors may account for the generalizability of overall models. One potential contributing element is the inherent heterogeneity of particular subgroups, which could arise from inconsistent data structures or haphazard questionnaire responses. To quantify the intra‐group heterogeneity, we separately built models for different subgroups, subsequently computing the intra‐group reconstruction error according to these specific models. Likewise, 100 random models were generated by randomly replacing labels and repeating the same procedures. Since models were constructed for specific subgroups themselves, a lower reconstruction error relative to that of the random models indicates less intra‐group heterogeneity compared to randomly selected subgroups of equal size (one‐sample t‐test). Here, the intra‐group heterogeneity of an individual subgroup was defined as the average “actual‐random” differences of reconstruction error when applying the population‐specific model. Furthermore, we examined additional influencing factors, including the model similarity and the sample size of the subgroups. The similarity between population‐specific models and overall models was delineated by the concordance index.

### 4.9 Prediction Analysis within the Big Two Space

With the aim of systematically assessing the link between personality traits and emotional, cognitive, and neural phenotypes, we developed a geometric framework for mapping these diverse measures onto a 2D Big Two space (see Supplementary Methods for details). The orthogonal axes of this space were defined by the Big Two traits identified in this study, allowing us to examine how different combinations of Big Two traits relate to brain and behavior patterns. We conceptualized each participant's personality profile as a position within this Big Two space and systematically examined 180 directional axes (from 0° to 180°) to capture all possible Big Two combinations. For each direction, we calculated a target score representing how strongly each participant expressed that particular trait orientation. This approach enabled us to identify which specific combinations of Big Two traits were most predictive of different phenotypic outcomes. Our analyses focused exclusively on the HCP‐YA participants with NEO‐FFI assessments because this cohort provided the necessary combination of standardized personality measures alongside comprehensive behavioral and neuroimaging data.

In particular, we employed thresholded partial least squares (T‐PLS) regression [29] to predict trait orientations from three phenotypic domains: emotional/psychiatric measures, cognitive assessments, and neural connectivity patterns. For each of the 180 directional axes, we trained separate predictive models using nested cross‐validation to ensure robust and unbiased performance estimates. The cross‐validation procedure respected family structure within the HCP dataset and included rigorous hyperparameter optimization (Figure S17). Statistical significance was assessed through permutation testing across all 180 directions with false discovery rate (FDR) correction for multiple comparisons. In order to identify which specific features consistently contributed to successful predictions, we used bootstrap resampling and estimated the stability of each predictor's contribution, generating Z‐scores that indicate both the strength and reliability of each feature's association with trait orientations [82]. Finally, we computed participant‐level signature scores by combining each individual's phenotypic profile with the most stable predictive features for each domain. These signatures provide interpretable indices that directly link multivariate brain and behavior patterns to specific orientations within the Big Two space, enabling transparent connections between personality structure and diverse phenotypic expressions.

### 4.10 Statistical Analysis

Data pre‐processing included reverse‐scoring of negatively keyed items, missing value interpolation, and standard fMRI denoising (e.g., motion correction, bandpass filtering, Fisher's z‐transformation). Data are presented as mean ± standard deviation (SD) for demographics, and median with interquartile range (IQR) for box plots. The total sample size was *n* = 1,336,840, with specific sub‐sample sizes (e.g., *n* = 988 for fMRI) detailed in Table 1 and figure legends. Statistical significance was assessed using two‐tailed one‐sample t‐tests, Pearson correlations, and permutation testing (5,000 permutations) for T‐PLS regression models. Feature stability was evaluated via 5,000‐sample bootstrap z‐statistics. Multiple comparisons were adjusted using the False Discovery Rate (FDR) method (*q* < 0.05). All statistical and machine learning analyses were performed using MATLAB.

## Author Contributions

K.Z., S.E., and D.V. conceptualized and designed the study. J.C., W.C., J.Q., and J.F. provided support in the original conception of this project. K.Z. and J.H. conducted data curation and validation. K.Z. performed data analysis, and drafted the manuscript, with subsequent revisions made by all authors.

## Conflicts of Interest

The authors declare no conflicts of interest.

## Supporting information




**Supporting File**: advs74732‐sup‐0001‐SuppMat.docx.

## Data Availability

The IPIP‐120 and IPIP‐300 inventories are constructed from IPIP items, and the response datasets analyzed in this study are publicly available from Johnson's OSF repository (https://osf.io/tbmh5/). NEO‐FFI questionnaire data, domain‐based behavior data, and neuroimage data obtained from the HCP datasets are publicly available at https://www.humanconnectome.org/. NEO‐PI‐R questionnaire data from the ESCS dataset is available at https://dataverse.harvard.edu/dataverse/ESCS‐Data. The BBC data is available at https://beta.ukdataservice.ac.uk/datacatalogue/doi/?id=7656#!#1.
