# Supplementary material for: Machine‐Learning Decomposition Identifies a Big Two Structure in Human Personality with Distinct Neurocognitive Profiles
Source: Adv Sci (Weinh). 2026 Mar 25;13(28):e09009. doi: 10.1002/advs.202509009 (PMC13185821; doi:10.1002/advs.202509009)
Supplement: Supplementary file 1 — Supporting File: advs74732‐sup‐0001‐SuppMat.docx. [file ADVS-13-e09009-s001.docx]

**Machine-Learning Decomposition Identifies a Big Two Structure in Human Personality with Distinct Neurocognitive Profiles**

Kaixiang Zhuang^1^, Ji Chen^2^, Jinfeng Han^3^, Wei Cheng^1^, Jiang Qiu^3^, Jianfeng Feng^1^, Simon Eickhoff^4,5^, Deniz Vatansever^1^*

*^1^ Institute of Science and Technology for Brain-Inspired Intelligence, Fudan University, Shanghai, China*

*^2^ Center for Brain Health and Brain Technology, Global Institute of Future Technology, Shanghai Jiao Tong University, Shanghai, China*

*^3^ School of Psychology, Southwest University, Chongqing, China*

*^4^ Institute of Neuroscience and Medicine, Brain and Behaviour (INM-7), Research Center Jülich, Jülich, Germany*

*^5^ Institute of Systems Neuroscience, Medical Faculty, Heinrich Heine University Düsseldorf, Düsseldorf, Germany*

**Address for Correspondence:**

Deniz Vatansever, PhD

Institute of Science and Technology for Brain-inspired Intelligence

Fudan University, Zhangjiang Campus

825 Zhangheng Road

Shanghai, PR China, 201203

E-mail: [deniz@fudan.edu.cn](mailto:deniz@fudan.edu.cn)

# Supplementary Methods

## OPNMF-based factor decomposition of personality traits

We used orthogonal projective non-negative matrix factorization (OPNMF) (Sotiras et al., 2015; Yang & Oja, 2010) to decompose Big Five questionnaire data into a set number of factors. Unlike traditional exploratory factor analysis or principal component analysis, OPNMF requires all elements in the matrix decomposition to be non-negative. This constraint allows us to identify clear, interpretable structures that represent core sources of variation and contribute additively to observed patterns. While OPNMF was first developed for applications in biology, image processing, and text mining, it excels at identifying latent components that combine additively rather than through mutual cancellation (Guo et al., 2024; Lee & Seung, 1999; Yang & Oja, 2010). This method has gained recognition in psychometric research for its ability to reveal interpretable, additive components that offer valuable insights into psychological constructs (Camilleri et al., 2021; Chen et al., 2020; Cheng et al., 2023).

The application of OPNMF to personality surveys has several distinctive features that offer important advantages (Berry et al., 2007; Sotiras et al., 2015):

(1) Parts-based representation

Traditional matrix decomposition methods, such as principal component analysis and factor analysis, generate global features through linear combinations of data with both positive and negative weights. However, this approach may not optimally represent personality traits, which typically comprise multiple interrelated yet distinct facet-like components. For example, Extraversion encompasses various sub-traits (or facets) such as sociability and assertiveness, each meaningfully contributing to the overall construct (Soto & John, 2017). The OPNMF approach, with its locality and non-negativity constraints (Lee & Seung, 1999), provides a more suitable framework for modeling these sub-traits. Moreover, OPNMF's constraints enable the identification of granular components within each scale while enhancing interpretability, as broader traits can be represented through linear weighted combinations of these constituent parts. Although individual components maintain non-negativity, it is important to note that negative correlations between traits may still emerge, aligning with established personality theory. Moreover, when constituent parts exhibit positive covariation that cannot be adequately captured by a single dimension, OPNMF preserves these discrete structures rather than forcing them into a single component.

(2) Orthogonal factor structure

A distinctive feature of OPNMF is how it implements orthogonality, which differs fundamentally from traditional factor analytic methods. In OPNMF, orthogonality is enforced among the factor loading vectors (columns of basis matrix *W*), ensuring each factor captures a mathematically independent pattern of item covariation. This constraint promotes interpretable, non-redundant factor structures where each dimension represents a distinct source of variation, facilitating clear item assignment and psychological interpretation. Importantly, while the factor loading vectors are mutually orthogonal, individuals' factor scores remain unconstrained and can correlate naturally. This design preserves realistic trait intercorrelations well-established in personality research, allowing the method to reveal genuine psychological relationships rather than imposing artificial independence among traits.

(3) Sparse components

OPNMF tends to yield sparse solutions through its optimization algorithm, resulting in factor structures where each personality dimension is defined by a subset of highly salient items while minimizing contributions from less relevant indicators. This data-driven sparsity reduces reliance on arbitrary threshold criteria often employed in traditional factor analyses, such as factor loading cutoffs (e.g., > 0.3) or relative loading comparisons, thereby providing a transparent basis for assigning items to factors. This sparsity enhances interpretability by offering a focused representation of item-factor relationships and attenuating noise from weak or potentially spurious item-factor associations.

(4) Generalizability

OPNMF’s projective form allows the learned basis to be directly applied to held-out data, enabling straightforward out-of-sample projection. While non-negativity and the tendency toward sparse loadings may act as implicit regularization, we treat generalizability as an empirical question and quantify it using cross-validation and increased reconstruction error (iRE).

(5) Flexible resolution of latent factors

A key advantage of OPNMF is its flexibility in factor dimensionality, allowing researchers to examine personality data at multiple levels of resolution. With fewer factors, the method identifies broad dimensions such as Big Two and Big Five traits. Conversely, with more factors it reveals nuanced sub-traits and facet-level distinctions. This adjustable rank decomposition provides personality psychologists with a unified framework for examining both global personality structure and fine-grained individual differences, eliminating the need for multiple traditional methods (Strus & Cieciuch, 2021). Furthermore, since general personality dimensions emerge from combinations of fine-grained components, this multi-scale decomposition facilitates exploration of the relationships between broad traits and their constituent facets (DeYoung et al., 2007), revealing how specific psychological characteristics combine to form broader patterns.

Mathematically, OPNMF is achieved by minimizing the reconstruction error (RE), measured by the squared Frobenius norm between the input data matrix $V$ and its low-rank estimate $W W^{T}V$, by iteratively updating the basis matrix $W$:

$${\min\left\| V- \right.\left. WW^{T}V \right\|}_{F}^{2}$$

$s.t. W^{T}W=I_{r}$,

where $W$ ($W\geq0$) is an $m$ (number of items) × $r$ (number of factors) matrix that conveys factor information in each column with respect to the co-occurrence properties of the questionnaire items. Entry $W_{ij}$ of $W$ is the loading of item $i$ on factor $j$. The columns of $W$ are orthonormal, i.e., $W^{T}W=I_{r}$. Under this constraint, $P= WW^{T}$ is an idempotent projection matrix onto the subspace spanned by the columns of $W$, onto which the data matrix $V$ can be projected in order to obtain an approximate estimate of itself. In this form, the loading matrix $H$ is replaced by $W^{T}V$, allowing the learned basis matrix $W$ to be used for representing new data. The orthogonality between the vectors in the learned $W$, combined with the non-negativity constraint and the multiplicative update scheme, tends to yield sparse factors, i.e., dimensions of personality traits that are defined by a relatively small number of highly weighted items in the current study.

$H$ can then be represented as a $r$ (number of the estimated factors) × $n$(number of the participants) matrix, encoding the factor scores of a given participant along the factors spanned by the basis matrix $W$. Entry $H_{jk}$ of $H$ represents the expression level of factor $j$ in participant $k$. The following multiplicative update rule was used to iteratively approach the non-convex problem, ensuring the positivity of the estimated factors while monotonically decreasing the energy towards a local optimum (Yang & Oja, 2010):

$W_{ij}\leftarrow W_{ij}\cdot\frac{\left( VV^{T}W \right)_{ij}}{\left( WW^{T}VV^{T}W \right)_{ij}}$.

The initialization strategy used here was nonnegative double singular value decomposition (NNDSVD) (Boutsidis & Gallopoulos, 2008), which has the advantage of lower residual error, faster convergence than random initialization, and most importantly, makes the final non-negative decomposition deterministic.

## Model evaluation indices

We developed a cross-validation-based evaluation scheme for OPNMF decomposition that fundamentally differs from traditional factor analysis approaches in both methodology and objectives (Chen et al., 2020). While conventional factor analysis primarily relies on model fit indices (e.g., CFI, RMSEA) and factor loading patterns within individual samples (Fabrigar et al., 1999), our approach prioritizes factor solution stability and generalizability across multiple samples. The evaluation framework integrates complementary indices of stability and generalizability. Structural stability was assessed using the Adjusted Rand Index (aRI) and Variation of Information (VI). Loading-pattern stability was quantified with the Concordance Index (CI). Cross-sample generalizability was evaluated using Increased Reconstruction Error (iRE). Finally, item-level stability was summarized with Item Variability (IV). These metrics offer distinct perspectives on factor solution quality, emphasizing stability and generalizability that are crucial for psychological measurement applications. The following sections provide detailed explanation of each metric's mathematical foundation and practical implementation:

1. Adjusted rand index

Rand index (RI) computes the similarity between two item-assignment results by considering all pairs of items and counting pairs that are assigned in the same or different factors between two decompositions:

$RI=\frac{number of agreeing pairs}{number of pairs}$.

The raw RI score is then adjusted for chance into the adjusted rand index (aRI) score, in which a correction for chance establishes a baseline by using the expected similarity $E\left( RI \right)$ of all pairwise comparisons between items specified by a random model (Hubert & Arabie, 1985):

$aRI=\frac{RI-E\left( RI \right)}{1-E\left( RI \right)}$.

Given a set $S$ of $n$ elements (items), and two item-assignment results, namely $C=\{C_{1},C_{2},C_{3},\cdots C_{r}\}$ and $C'=\{{C'}_{1},{C'}_{2},{C'}_{3},\cdots{C'}_{s}\}$, the overlap between $C$ and $C'$ can be summarized in a contingency table $n_{ij}$ where each entry indicates the number of objects in common between $C_{i}$ and ${C'}_{j}$. The expected similarity can be identified as:

$E\left( RI \right)=\frac{\sum_{i} \binom{a_{i}}{2}}{\binom{n}{2}}\cdot\frac{\sum_{j} \binom{b_{j}}{2}}{\binom{n}{2}}+\left( 1-\frac{\sum_{i} \binom{a_{i}}{2}}{\binom{n}{2}} \right)\cdot\left( 1-\frac{\sum_{j} \binom{b_{j}}{2}}{\binom{n}{2}} \right)$,

where $n_{ij}$, $a_{i}$ and $b_{j}$ are the values from the contingency table: $n_{ij}=$|$C_{i}\cap{C'}_{j}$|; $a_{i}=\sum_{j} n_{ij}$; $b_{j}=\sum_{i} n_{ij}$.

1. Variation of information

Variation of information (VI), also known as shared information distance (Meilă, 2003) is used to quantify the distance between two item-assignment results, that can be identified as:

$VI\left( C,C' \right)=H\left( C \right)+H\left( C' \right)-2I\left( C;C' \right)$,

where $H\left( C \right)$ and $H\left( C' \right)$ indicate the marginal entropies (amount of information) in item-assignment results of $C$ and $C'$, and ${I\left( C;C' \right)}_{K}$ represents how much mutual information is contained between them, which can be defined as:

$I\left( C;C' \right)=\sum_{k=1}^{K} \sum_{k'=1}^{K'} P\left( k,k' \right)\cdot log\frac{P(k,k')}{P(k)\cdot P\left( k' \right)}$,

and

$H\left( C \right)=-\sum_{k=1}^{K} P\left( k \right)\cdot{log}P\left( k \right)$,

where $P(k)$ and $P\left( k' \right)$ are the probability that an item belongs to a factor $k$ or $k'$, respectively. $P(k,k')$ is the probability that an item belongs to factor $k$ in $C$ and factor $k'$ in $C'$. A VI of 0 indicates that there is complete overlap between two item-assignment results, and higher values indicate lower similarity between them.

1. Concordance index

Apart from the clustering-like factor structure (i.e., hard-assigned items), OPNMF produces a basis matrix $W$ that assigns continuous nonnegative weights to each item across factors. We quantified the stability of these item weights across two OPNMF decompositions using the concordance index (CI)(Raguideau et al., 2016). For a given decomposition, let $W\in\mathbb{R}_{+}^{m\times r}$ denote the basis matrix with $m$ items (rows) and $r$ factors (columns).

First, $W$ was normalized row-wise to obtain $\tilde{W}$ by dividing each row by its Euclidean norm:

$\tilde{W}_{ij}=\frac{W_{ij}}{\left( \sum_{j^{'}=1}^{r} W_{ij^{'}}^{2} \right)^{1/2}}$, $i=1,...,m, j=1$,…,*r*.

Next, a symmetric item–item similarity matrix $S^{\tilde{W}}\in\mathbb{R}_{+}^{m\times m}$ was constructed as the cosine similarity between item-loading vectors (rows) of $\tilde{W}$, which reduces to a dot product after row normalization:

$S^{\tilde{W}}={\tilde{W}\tilde{W}}^{T}$, $S_{ii^{'}}^{\tilde{W}}=\sum_{j=1}^{r} \tilde{W}_{ij}\cdot\tilde{W}_{i^{'}j}$.

Because each row of $\tilde{W}$ has unit norm, $S_{ii}^{\tilde{W}}=1$ for all $i$*.* Given two decompositions with normalized basis matrices $\tilde{W}^{(1)}$ and $\tilde{W}^{(2)}$, CI was defined as one minus the mean squared difference between the corresponding similarity matrices (effectively averaging over off-diagonal entries):

$CI=1-\frac{\parallel S^{\tilde{W}^{(1)}}-S^{\tilde{W}^{(2)}}\parallel_{F}^{2}}{m^{2}-m}$.

1. Increased reconstruction error

The reconstruction error (RE) was computed as the participant-averaged sum of absolute reconstruction residuals across items between the original data matrix and its low-rank approximation (Chen et al., 2020). Specifically, given a basis matrix $W$, the data matrix $V$ was reconstructed as $\hat{V}\boldsymbol{=}{WW}^{T}V$. For each participant, absolute residuals $\left| \text{round}\left( \hat{V}_{ik} \right)-\left( V \right)_{ik} \right|$ were summed across items, and RE was obtained by averaging these sums across participants. Smaller RE indicates better reconstruction by a given basis matrix $W$. Within-sample RE was computed using the basis estimated from the same sample, whereas cross-sample RE was computed by applying the basis estimated from the training sample to reconstruct the hold-out sample. Increased reconstruction error (iRE) was defined as the mean absolute difference between cross-sample and within-sample participant-level reconstruction errors and was used as the final index of generalizability.

In detail, applying the OPNMF, the basis matrices $W_{1}$ and $W_{2}$ were separately generated for two submatrices $V_{1}$ and $V_{2}$. Then the two submatrices were reconstructed as:

$\hat{V}_{1|1}=W_{1}{W_{1}}^{T}V_{1}$,

and

$\hat{V}_{2|2}=W_{2}{W_{2}}^{T}V_{2}$,

The within-sample reconstruction error can be identified as:

$RE_{1}=\frac{1}{n_{1}}\sum_{k=1}^{n_{1}} \sum_{i=1}^{m} \left| \text{round}\left( \left( \hat{V}_{1|1} \right)_{ik} \right)-\left( V_{1} \right)_{ik} \right|$,

and

$RE_{2}=\frac{1}{n_{2}}\sum_{k=1}^{n_{2}} \sum_{i=1}^{m} \left| \text{round}\left( \left( \hat{V}_{2|2} \right)_{ik} \right)-\left( V_{2} \right)_{ik} \right|$.

The cross-sample reconstruction from $V_{1}$ using the basis $W_{2}$ learned from $V_{2}$ is:

$\hat{V}_{1|2}{=W}_{2}{W_{2}}^{T}V_{1}$,

and the corresponding cross-sample reconstruction error is:

${\hat{\mathrm{RE}}}_{1|2}= \frac{1}{n_{1}}\sum_{k=1}^{n_{1}} \sum_{i=1}^{m} \left| \text{round}\left( \left( \hat{V}_{1|2} \right)_{ik} \right)-\left( V_{1} \right)_{ik} \right|$.

Finally, the increased reconstruction error from $V_{2}$ to $V_{1}$ is formulized as:

$iRE_{2\to1} =\frac{1}{n_{1}}\sum_{k=1}^{n_{1}} \left| \sum_{i=1}^{m} \left| \text{round}\left( \left( \hat{V}_{1|2} \right)_{ik} \right)-\left( V_{1} \right)_{ik} \right| - \sum_{i=1}^{m} \left| \text{round}\left( \left( \hat{V}_{1|1} \right)_{ik} \right)-\left( V_{1} \right)_{ik} \right| \right|$,

where $n_{1}$ and $n_{2}$ denote the numbers of participants in $V_{1}$ and $V_{2}$, respectively.

1. Item variability

To further detect the fallibilities of items during cross-validation, an item level metric of variation was also applied in the current study (Steen et al., 2011). For each item, the item variability (IV) can be identified as:

${IV}_{k}=1-\frac{\left| X_{k}\cap Y_{k} \right|}{\left| X_{k} \right|}\cdot\frac{\left| X_{k}\cap Y_{k} \right|}{\left| Y_{k} \right|}$,

where $\left| X_{k} \right|$ and $\left| Y_{k} \right|$ denote the number of items having the same factor affiliation with item $k$ (including $k$ itself) in the item-assignment results of $X$ and $Y$, respectively; $\left| X_{k}\cap Y_{k} \right|$ represents the number of items in the common item sets of $X$ and $Y$. Thus, the small overlap between $X_{k}$ and $Y_{k}$ leads to large factor affiliation variability of item $k$.

## Cross-validation strategies

The main model evaluation was implemented through 5-fold cross-validation (5F-CV), in which samples were split into 5 folds, retaining 4/5 of the main sample as training set, and 1/5 hold-out sample as test set. In both the main and hold-out samples, the OPNMF-based factor decomposition was applied, generating a clustering-like factor structure (i.e., the hard-assigned items) and the basis matrix that specifies items’ contributions to each factor. Subsequently, the model stability evaluation indices (i.e., aRI, VI, CI and IV) were calculated based on two factor structures and basis matrices between the main sample and the hold-out sample. In addition, the hold-out sample data matrix was projected to the basis matrix obtained from the main sample by calculating the dot products between these two matrices. The model generalizability index (i.e., iRE) was then generated based on the comparison between out-of-sample RE and the within-sample RE. The above process was repeated for five times to ensure that each of the folds has been treated as the hold-out sample once. And the obtained values were averaged over the five repetitions as the final metrics for one 5F-CV implementation.

In addition to 5F-CV, bootstrap approach was also applied while implementing the cross-sample or cross-version evaluations. When compared to 5F-CV, the bootstrap pipeline differed primarily in how the main and hold-out samples were generated. We bootstrapped samples from different data sets independently in each implementation of the bootstrap-based comparison between two samples, then assigned one of the resulting bootstrapped samples as the main sample and the other as the hold-out sample. Afterwards, the above-mentioned evaluation indices (i.e., aRI, VI, CI, IV and iRE) were calculated between two bootstrapped samples in the same way as in the 5F-CV pipeline.

The two pipelines described above were used in all model evaluations in the current study, but they were subject to variations according to data characteristics. Due to the randomness of resampling, all evaluations were iterated 1,000 times, resulting in 1,000 sets of randomly assigned 5-folds or bootstrapped samples. In order to avoid the influence of outliers, we set the final value of each evaluation index to the median of 1,000 iterations.

## Cross-validation with additional information considered

To examine the robustness of the model in the face of additional perturbations, we also considered the potential influence of factors such as culture (i.e., countries/regions), item version (i.e., brief version and full version), and questionnaire type (i.e., NEO-FFI and IPIP-NEO) while performing cross-validation for both the IPIP-NEO response data and the NEO-FFI response data.

To examine the stability and generalizability of the model across cultures for IPIP-120, we used 1,000 culture-based 5F-CV, that respected the culture effects by never splitting data in the same countries/regions between folds. Given that about 87.0% of the data in IPIP-120 originated from four countries (N in US = 447,500; N in CA = 36,874; N in UK = 31,236; N in AU = 22,658), an additional resampling method was performed that randomly extracts 1,000 data points from each of these four countries separately before each implementation of the 5F-CV. After resampling, these four countries only accounted for about 4.7% of the total data in each cross-validation, which amplifies the influence of data from under-represented countries/regions for model evaluation.

To assess the possible impact of item versions, we employed a 1,000 cross-sample bootstrap method on the shared items in both IPIP-120 and IPIP-300, as well as between NEO-FFI and NEO-PI-R. That is, in each bootstrap iteration, models built within IPIP-120 were generalized to the bootstrapped data in IPIP-300, to test the fitness of the model for different questionnaire versions. Similarly, in each loop of the model evaluation, models built in NEO-FFI were also applied to the bootstrapped data in NEO-PI-R.

Moreover, an additional model evaluation was carried out between NEO-FFI and IPIP-NEO. To achieve this, we randomly selected an equal number of participants from the IPIP-120 response data for each cross-validation loop to match the sample size of the HCP dataset (*n* = 2,152), and applied the models built in NEO-FFI to the randomly extracted data in IPIP-NEO. Despite the non-identical nature of the items across the two questionnaires, certain facets (as previously mentioned) are shared between them, which enable cross-questionnaire evaluation. As a result, we calculated the weighted average of the items attributed to each shared facet and obtained scores for the 26 shared facets in both questionnaires. Subsequently, we performed cross-validation based on the shared facets rather than the original items.

Taking into account factors such as questionnaire version and sample variation (e.g., differences among samples in HCP datasets), we carried out 7 evaluations for IPIP-NEO and 10 evaluations for NEO-FFI, each employing a variety of methodological strategies.

## All evaluations of factor decomposition within the IPIP-NEO response data

1. Five-fold cross-validation (5F-CV) in IPIP-120 (basic evaluation):

The evaluation encompassed the entire IPIP-120 response data. Missing values were imputed using the mode of the corresponding item. Further details are provided in the main manuscript.

1. Culture-based 5F-CV in IPIP-120 (evaluation considering culture):

This evaluation also included the entire IPIP-120 response data, annotated with 240 country/region labels. Missing values were handled identically to the basic evaluation. Additional information is available in the main manuscript.

1. Cross-sample bootstrap-based evaluation between IPIP-120 and IPIP-300 (evaluation considering version):

The evaluation spanned the full IPIP-120 and IPIP-300 response data. Missing values were addressed as in the previous evaluations. The main manuscript contains detailed descriptions.

1. 5F-CV in the complete IPIP-300:

The same evaluation pipeline used in the basic evaluation was applied to the complete IPIP-300 response data, which includes all 300 items.

1. 5F-CV in the 120 shared items from IPIP-300:

The evaluation protocol from the basic evaluation was utilized for IPIP-300 data, but only the 120 items shared with IPIP-120 were considered.

1. Culture-based 5F-CV in complete IPIP-300:

The evaluation pipeline employed in the culture-based evaluation was also applied to the IPIP-300 response data, encompassing all 300 items.

1. Culture-based 5F-CV in the 120 shared items from IPIP-300

The culture-based evaluation protocol was applied to the IPIP-300 data, focusing only on the 120 items that overlap with IPIP-120.

The outcomes of all the above evaluations are shown in the Extended Data Table 1.

## All evaluations of factor decomposition within the NEO-FFI and NEO-PI-R response data

1. 5F-CV in the NEO-FFI data from the integrated HCP dataset (basic evaluation):

The evaluation was conducted using NEO-FFI data from the integrated HCP dataset, comprising three cohorts (HCP-D, HCP-YA, and HCP-A). Missing values in HCP-D and HCP-A were imputed using the mode of the respective item.

1. Cross-sample bootstrap-based evaluation between NEO-FFI and NEO-PI-R (evaluation considering version):

This evaluation involved NEO-FFI data from the integrated HCP dataset and NEO-PI-R data from the ESCS dataset. In each evaluation loop, models built on NEO-FFI data were applied to bootstrapped samples from NEO-PI-R.

1. Cross-sample bootstrap-based evaluation between NEO-FFI and IPIP-120 (evaluation considering questionnaire):

The evaluation focused on the 26 shared facets or subdimensions between NEO-FFI and IPIP-120. Items were averaged according to their respective facet in both NEO-FFI and IPIP-120, encompassing all available data. For each of the 1,000 iterations, an equivalent number of participants to the HCP dataset (i.e., *n* = 2,152) were randomly sampled from the IPIP-120 data, and models constructed from NEO-FFI data were applied to these samples. Despite the items not being identical across the two questionnaires, their shared facets enabled cross-questionnaire evaluation.

1. Family-based 5F-CV in the NEO-FFI from the HCP-YA dataset:

A similar evaluation pipeline to that of the basic evaluation was applied. However, was exclusively conducted on NEO-FFI data from the HCP-YA dataset. This evaluation accounted for family effects by ensuring that data from the same family were not divided between different folds.

1. 5F-CV in the NEO-FFI from the HCP-A dataset:

The evaluation mirrored the basic evaluation but was confined to NEO-FFI data from the HCP-A dataset.

1. 5F-CV in the NEO-FFI from the HCP-D dataset:

This evaluation also followed the basic evaluation protocol, focusing solely on NEO-FFI data from the HCP-D dataset.

1. Cross-sample bootstrap-based evaluation across three datasets

A similar evaluation pipeline to that considering version differences was employed. In this evaluation, models built on NEO-FFI data from the HCP-YA dataset were applied to bootstrapped samples from the HCP-A and HCP-D datasets.

1. 5F-CV in the complete NEO-PI-R data from ESCS dataset

The evaluation pipeline used in the basic evaluation was also applied to the complete NEO-PI-R dataset, which comprises all 240 items.

1. 5F-CV in the 60 shared items from NEO-PI-R data:

The evaluation approach from the basic evaluation was used for the 60 items common to NEO-PI-R.

1. Cross-sample bootstrap-based evaluation between NEO-PI-R and IPIP-120:

The evaluation pipeline that takes into account the questionnaire was applied to both NEO-PI-R and IPIP-120, which share 30 facets or subdimensions based on the five-factor model of personality.

The outcomes of all the above evaluations are shown in the Extended Data Table 1.

## Semantic alignment of questionnaire items across instruments

Given the novelty of the Big Two model, we tested whether versions derived from different questionnaires capture the same underlying psychological construct. To do so, we compared item-level semantic similarity across instruments for each factor. Higher similarity within a given factor would indicate that the Big Two structure represents a comparable construct across instruments. Specifically, we aligned items across two instruments through semantic similarity matching enhanced by lexical overlap. First, we preprocessed both instruments by converting negatively-worded items to affirmative form and appending dimension labels as contextual cues. Each item was then encoded into a 1,024-dimensional vector using the BAAI/bge-m3 sentence-embedding model (Chen et al., 2024), implemented via the Transformers library (Wolf et al., 2020), with mean pooling over token representations (Reimers & Gurevych, 2019) and L2 normalization. We computed a full cosine similarity matrix between all cross-questionnaire item pairs as the baseline measure of semantic correspondence.

To better capture lexical evidence, we applied a token-overlap weight to each pairwise similarity. This weight boosted similarity when two items shared salient words or close synonyms. The token-overlap weight was derived from the Jaccard overlap between normalized token sets (after lowercasing, removing punctuation and stopwords, lemmatization, and synonym mapping), computed as 1.00 + 0.20·s and capped at 1.12. The final similarity score for each pair was the product of the cosine similarity and the token-overlap weight. For each source item, the target item with the highest final score was selected as its match. Each NEO-FFI and BFI item was then matched to its most semantically similar counterpart in the IPIP-120, and, within the NEO-FFI, to the best-matching items in the BFI.

## Community structures present within the association networks of culturally-bound personality models

The observed differences in personality models across various countries and regions are more pronounced than those associated with demographic factors. In an effort to explore potential adaptations of personality models across diverse cultural contexts, we examined the community structures present within the association networks of culturally-bound personality models. Here, we primarily examined the assortative and core-periphery structures (Betzel, Medaglia, et al., 2018). The assortative structure provides insight into the ways in which culture shapes personality structures, with countries/regions from the same cultural sphere (i.e., community) tending to have more similar personality structures, while differences in personality structures across cultural spheres are relatively large. The core-periphery structure reveals a phenomenon of convergence in personality structures, wherein select countries/regions exhibit representative personality structures while the personality structures of other countries/regions tend to approximate them.

To assess these structural attributes, we initially executed community detection on the association networks of the focused personality models among countries/regions. In this case, the nodes of the association network represent personality models specific to various countries/regions, while the edges depict the pairwise similarities between those models as quantified through CI. Here, the association networks were first thresholded at a range of edge densities ranging from 20% to 50% at 5% intervals. At every density threshold, we utilized the weighted stochastic blockmodel (WSBM), a pliable community detection technique, to uncover the meso-scale architecture of a given network (Aicher et al., 2013, 2015). Unlike the methods that maximize modularity index, the resulting communities of WSBM reflect similarities in nodes’ connectivity profiles, hence are not constrained to be assortative. We varied the number of communities from two to four, while setting the tuning parameter $\alpha\in[0,1]$ at 0.5 that balances the relative importance between edge weight and edge presence (or absence) for inference, and repeated the WSBM procedure 100 times. A consensus community structure was identified as the single representative structure that is most similar (as quantified by VI) to the all others. After conducting these operations across each edge density threshold, we applied an 'association-recluster' strategy, which records the frequency of nodes co-occurring in a community across an ensemble of partitions, to produce the final community detection results (Lancichinetti & Fortunato, 2012).

After each node was assigned to specific communities by the WSBM, we characterized the interactions between communities by examining their pairwise community densities through network morphospace analysis (Avena-Koenigsberger et al., 2015). Following that, we sorted each node into three motif classes in each WSBM implementation: assortative, disassortative and core (Betzel, Bertolero, et al., 2018; Betzel, Medaglia, et al., 2018). Given two pairwise communities ($i$ and $j$), a motif is deemed assortative if its minimum within-community density exceeds the between-community density. Conversely, a motif is considered disassortative if its maximum within-community density is deemed lower than the between-community density. If the within-community density of community $i$ surpasses that of the between-community density, and the latter exceeds the within-community of community $j$, then the nodes within community $i$ are classified as core, while those within community $j$ are identified as periphery. We carried out the aforementioned classifications across each edge density threshold and WSBM implementations. By averaging the number of times nodes were assigned to different motif classes, we extracted three node-level metrics: assortativity, disassortativity and coreness. Greater assortativity (or lower disassortativity) implies that the association network of personality models is relatively segregative, influenced by various cultural constraints. Whereas higher coreness suggests the presence of a core-periphery structure, and the personality models from different countries/regions tend to converge towards specific archetypal models.

## Prediction analysis within the Big Two space

Utilizing thresholded partial least squares (T-PLS) regression models (Lee et al., 2022), we aimed to map emotional/psychiatric, cognitive, and neural phenotypes onto directions within a two-dimensional factor space spanned by Social Adaptation (SA; 0° axis) and Spontaneous Mentation (SM; 90° axis). Each participant’s position was represented by a 2D vector *v_i_* = (*SA_i_*, *SM_i_*). For any direction *θ* ∈ [1°, 180°], the scalar target *y_i_*(θ) was defined as the signed projection of v_i_ onto the unit vector *uθ* = (cos θ, sin θ), i.e., *y_i_*(θ) = *SA_i_* cos *θ* + *SM_i_* sin *θ*. Thus, higher *y_i_*(*θ*) indicates stronger expression of the personality trait combination aligned with *θ*. We sampled *θ* over unique axes (0–180°); extending to 0–360° is equivalent but redundant (*θ* and *θ* + 180° define the same axis with opposite sign). Analyses were restricted to HCP-YA with NEO-FFI because this cohort provides large‑scale, harmonized Big Five assessments alongside matched behavioural and neuroimaging phenotypes, enabling in‑sample derivation of personality trait targets. In contrast, other questionnaires and datasets lacked comparable, co-registered phenotypic measures, rendering them unsuitable for this mapping.

For each *θ* we trained a separate T‑PLS model with nested five‑fold cross‑validation (outer 5F-CV for unbiased accuracy; inner 5F‑CV for hyperparameter tuning), while respecting family structure by ensuring relatives were assigned to the same fold. In the outer cross-validation procedure, the algorithm was trained on approximately 80% of the participants and tested on the remaining 20%, iterating across five folds to ensure each subset was tested once. During each fold, a linear model was used to adjust for the effects of age, sex, and head motion on each feature in the training dataset. The coefficients derived from the training data were then applied to remove these covariate effects from the corresponding features in the testing dataset. Additionally, the features in the training data were normalized to a range of 0 to 1, and the same scaling parameters were applied to the testing data for consistency. Importantly, the cross-validation process accounted for family-related effects in the HCP-YA dataset by ensuring that data from the same family were not split across different folds.

The two tuning parameters, which include the number of PLS components ranging from 1 to 25, and the threshold level varying from 0.1 to 1 (in increments of 0.1), were optimized based on the model's performance (*r*) during the inner 5F-CV of each respective training dataset (Figure S16). Utilizing the optimal parameters, models were trained on the entirety of the training data to predict the target variables within the testing dataset. By averaging the parameter performances across all iterations, we also determined the optimal parameters at the mean level. It is worth noting that, due to the limited number of features associated with behavioural phenotypes, we opted not to sparsify the feature set, instead setting the threshold level to 1.

We measured predictive accuracy using the Pearson's correlation coefficient (*r*) and the explained variance score (*r*²) between actual and predicted values for the model (Dubois et al., 2018). To enhance the stability of the outcomes, we executed 30 iterations of cross-validation, with the accuracy scores being averaged across all runs. Statistical significance of predictive performance for each *θ* was assessed via permutation testing with 5,000 iterations within the outer-CV framework: in each iteration, target values *y_i_*(*θ*) were randomly permuted within folds to preserve fold structure, the model was retrained with the hyperparameter tuning procedure, and the null distribution of *r* was obtained. Empirical *p* values were derived as the proportion of null *r* exceeding the observed mean *r*; false discovery rate was controlled across 180 directions using Benjamini–Hochberg false discovery rate (FDR) at *q* < 0.05.

To identify stable phenotype contributors, we performed bootstrap estimation (Kohoutová et al., 2020) of T‑PLS coefficients. For each *θ* and domain-specific model, we generated 5,000 bootstrap samples by resampling participants with replacement from the full dataset, re-estimated the T‑PLS model using the mean‑optimal hyperparameters, and computed Z scores for each predictor from the bootstrap coefficient distribution (Z = mean/SD). Two‑tailed *p* values were obtained from Z under a normal approximation and FDR‑corrected within model. The sign of Z indicates the direction of association between the predictor and increased expression along *θ*, while |Z| reflects contribution stability.

Finally, to derive participant-level composite expressions that can be correlated with Big Two and Big Five domains, we computed signature responses by taking the dot product between each participant’s preprocessed feature vector and the corresponding bootstrap Z‑score vector for the peak‑θ model within each domain. These signatures yield scalar indices of emotional/psychiatric, cognitive, and neural patterns most predictive of orientation in the Big Two space, thereby providing a transparent link between personality positioning and multivariate phenotypic profiles.

# Supplementary Figures


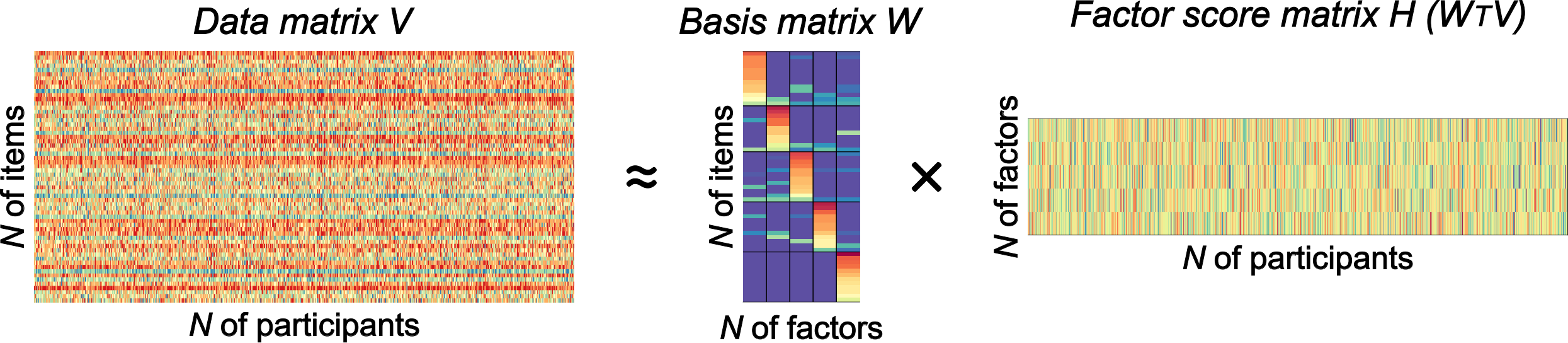


**Figure S1. Illustration of the OPNMF factorization.** OPNMF decomposes Big Five questionnaire data into two non-negative matrices: (1) a factor loading matrix (basis matrix), where the columns represent personality traits or factors that are highly interpretable due to the sparsity enforced by the orthonormality constraint and generalizable to new data because of the projective constraint, (2) A factor-score matrix, where the columns correspond to individual participants, representing their scores on each factor.


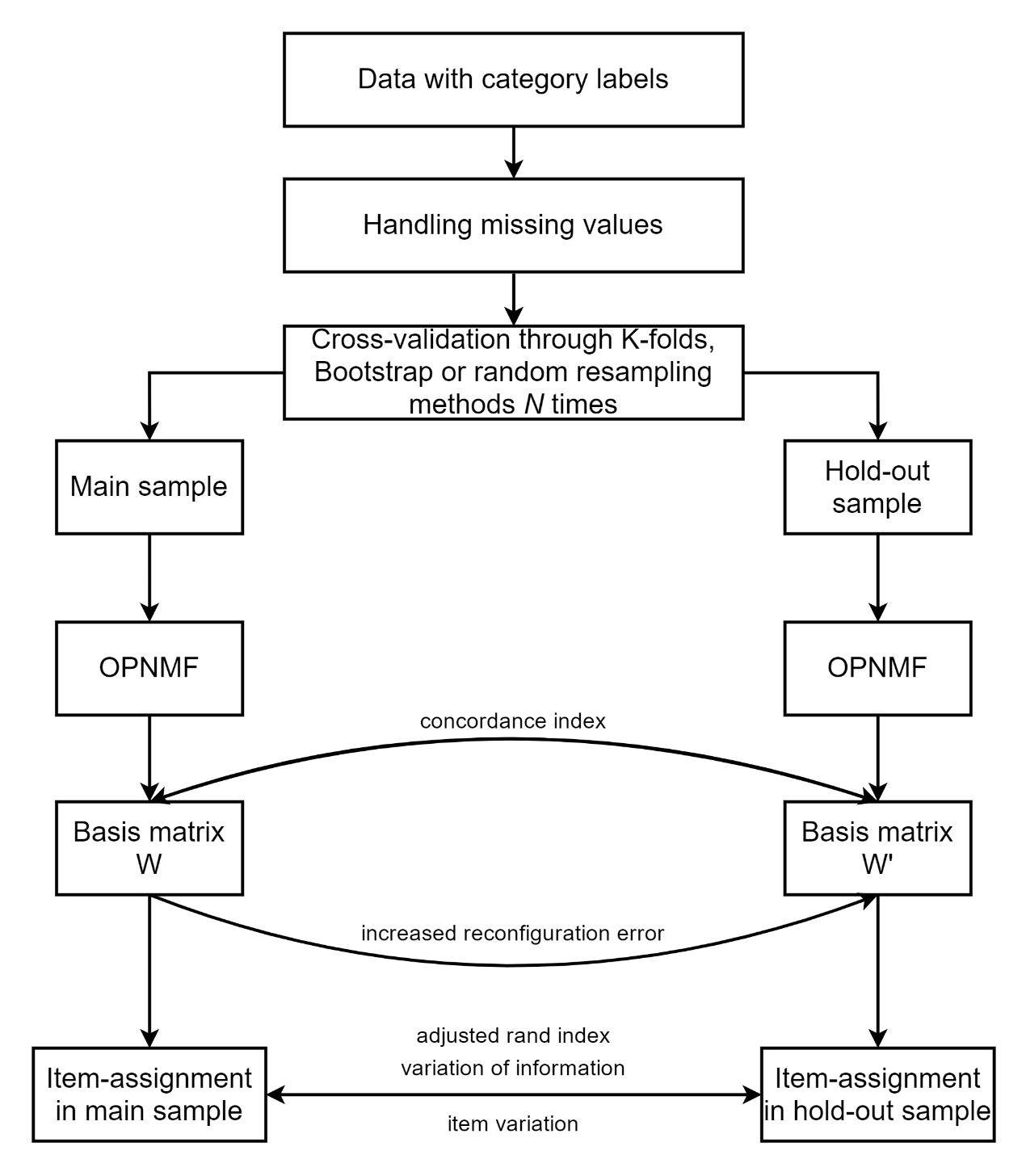


**Figure S2. General evaluation framework for OPNMF-based** **personality trait decomposition.** Following estimation of missing values using the modal response for each item, the Big Five questionnaire data (including categorical labels such as questionnaire version and data source) are split into a main sample and a hold-out sample using cross-validation strategies. OPNMF is then independently applied to the main and hold-out samples, and a series of evaluations is conducted to assess the resulting factor models. The direction of the arrows indicates the flow of information through the analysis pipeline.


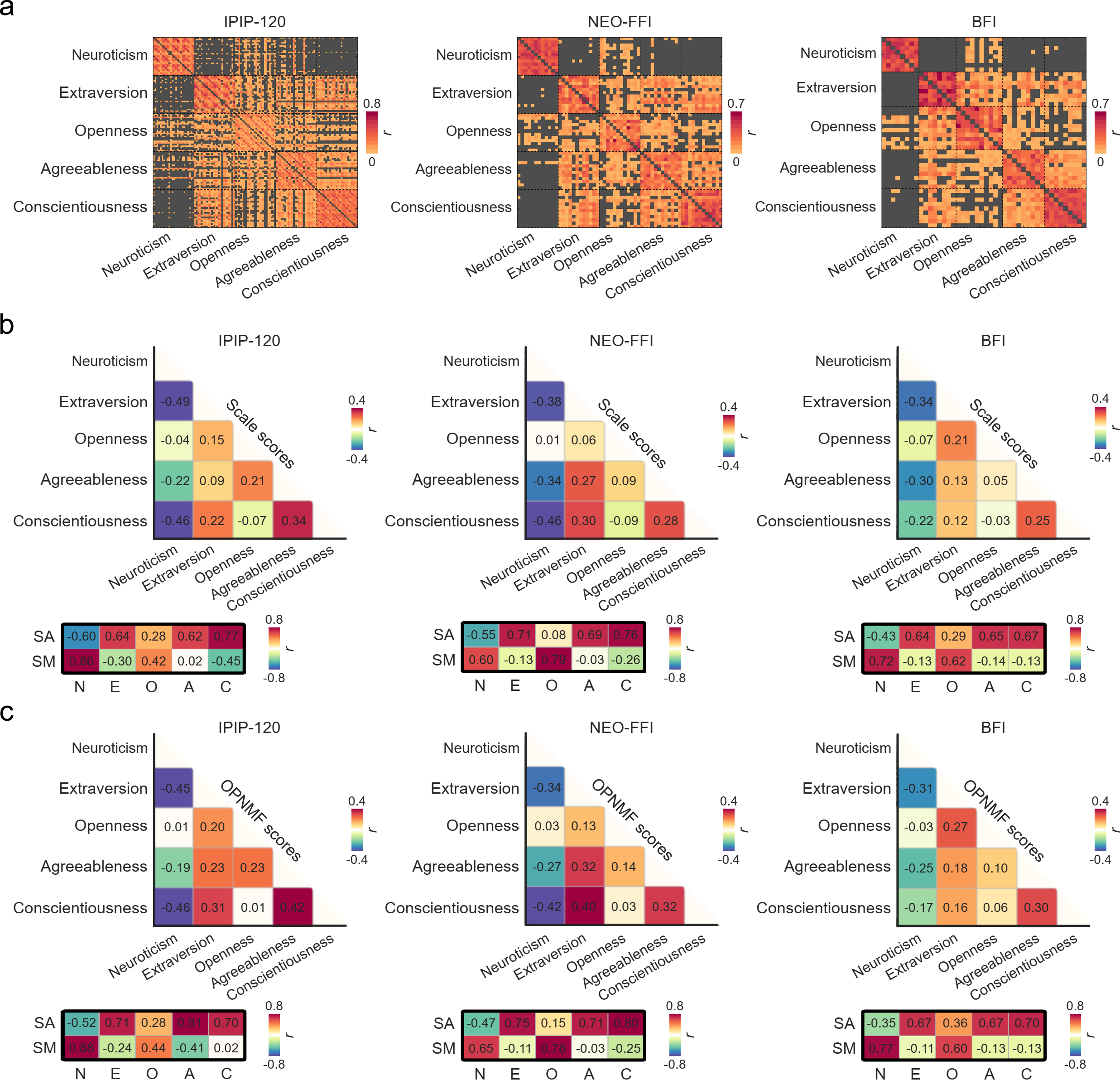


**Figure S3. Item-level and dimension-level correlations in Big Five personality questionnaires. a.** Positive correlations among items across three versions of the questionnaire (IPIP-120, *n* = 619,150; NEO-FFI, *n* = 2,152; BFI, *n* = 386,375). Edges between items represent Pearson correlation coefficients, with only positive correlations displayed. Items are arranged according to their theoretical Big Five dimensions. **b.** Correlations among original Big Five scores across the three questionnaires. Dimension scores are computed as weighted averages of item scores within each theoretical dimension for each instrument. **c.** Correlations among OPNMF-based dimension scores across the three questionnaires. In OPNMF, dimension scores are given by the corresponding factor scores. The upper heatmap shows correlations among the OPNMF-derived Big Five dimensions, and the lower heatmap shows correlations between the OPNMF-derived Big Two and the Big Five dimensions. The correlations between Social Adaptation (SA) and Spontaneous Mentation (SM) across the three inventories were -0.13 (for IPIP-120), -0.16 (for NEO-FFI), and -0.08 (for BFI).


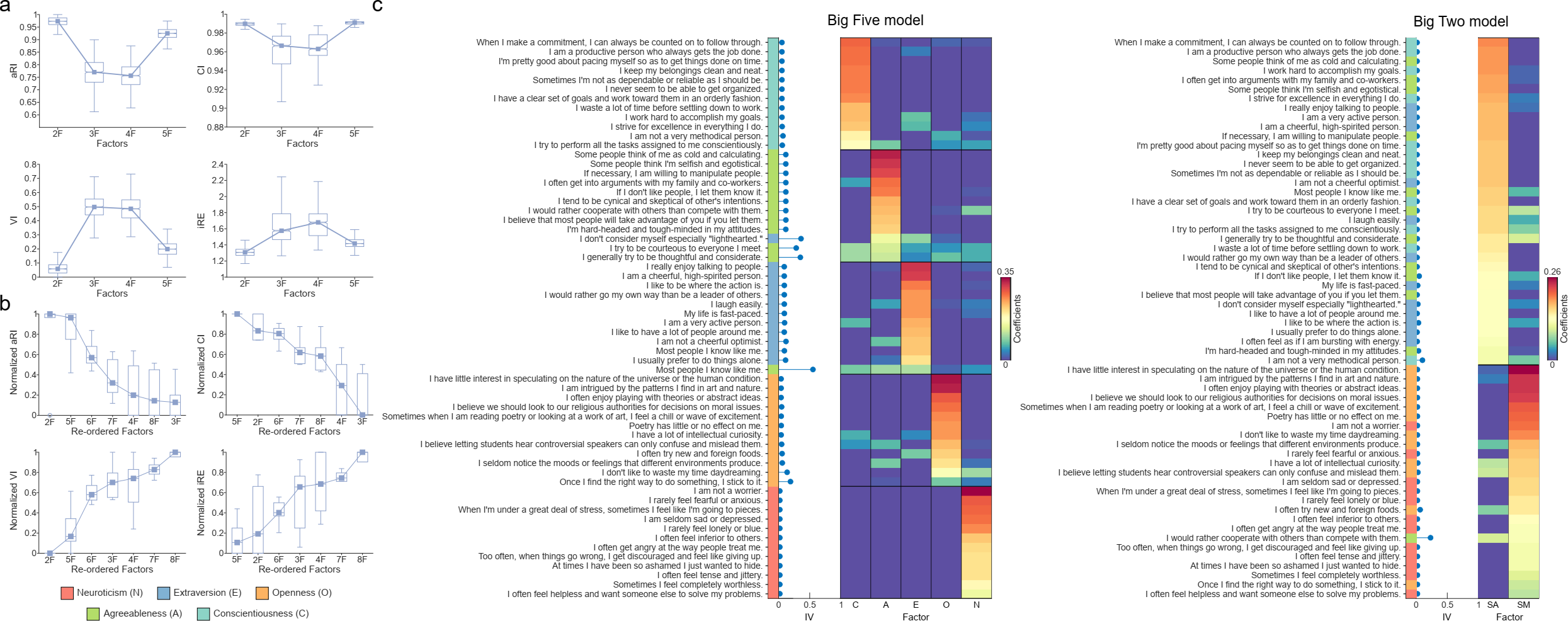


**Figure S4. Evaluation of personality trait decomposition for the NEO-FFI.** **a.** Main evaluation of model stability and generalizability in the HCP dataset (including HCP-YA and HCP-A/D). The 5F-CV derived evaluation indices, are employed to assess the robustness of item assignments in the NEO-FFI. Higher aRI and lower VI indicate greater consistency of item assignments across different OPNMF implementations, while higher CI and lower iRE indicate higher similarities between different basis matrices generated by OPNMF. The factor numbers (2F-5F) represent the number of factors considered. **b.** Summary of evaluation results. Box plots present the data points of four evaluation indices across a broader range of factor numbers (2F-8F) for the NEO-FFI. The factor numbers are re-ordered based on their evaluation performances. The complete set of evaluations is provided in Supplementary Table 2. **c.** Personality trait decomposition for the NEO-FFI. Items from the NEO-FFI are assigned to either the two factor or five factor models based on their maximum loading coefficients in the basis matrix generated from the entire NEO-FFI data in the HCP dataset. The IV index reflects the extent of variability exhibited by items during cross-validation, indicating the potential for items to change their assignments in the basic evaluation.


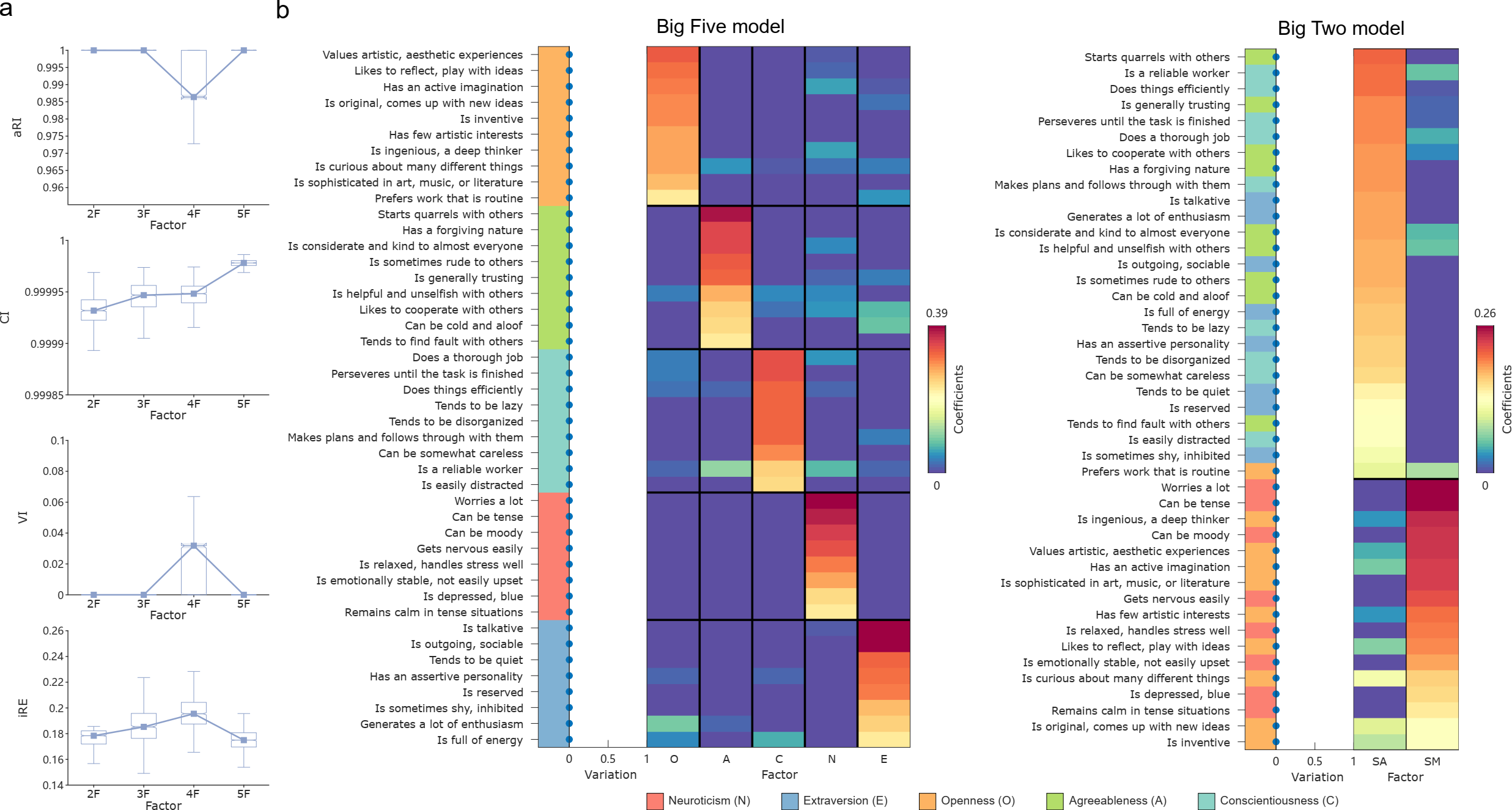


**Figure S5. Evaluation of personality trait decomposition in the BBC dataset.** **a.** Evaluation of model stability and generalizability. Evaluation of factorizations for two to five factor numbers are derived from the BBC dataset based on four evaluation indices. Higher aRI and lower VI linked to higher consistency of item affiliations between different OPNMF implementations; higher CI and lower iRE indicate higher similarities between different basis matrices from OPNMF. 2F-5F indicate factor numbers. **b.** Personality trait decomposition of the BFI in the BBC dataset. Items are assigned into two factor and five factor models according to their maximal loading coefficients of the basis matrix generated from the entire BBC data. Because there is no fluctuation of item affiliations within the 5F-CV, IV values all turn out to be 0.


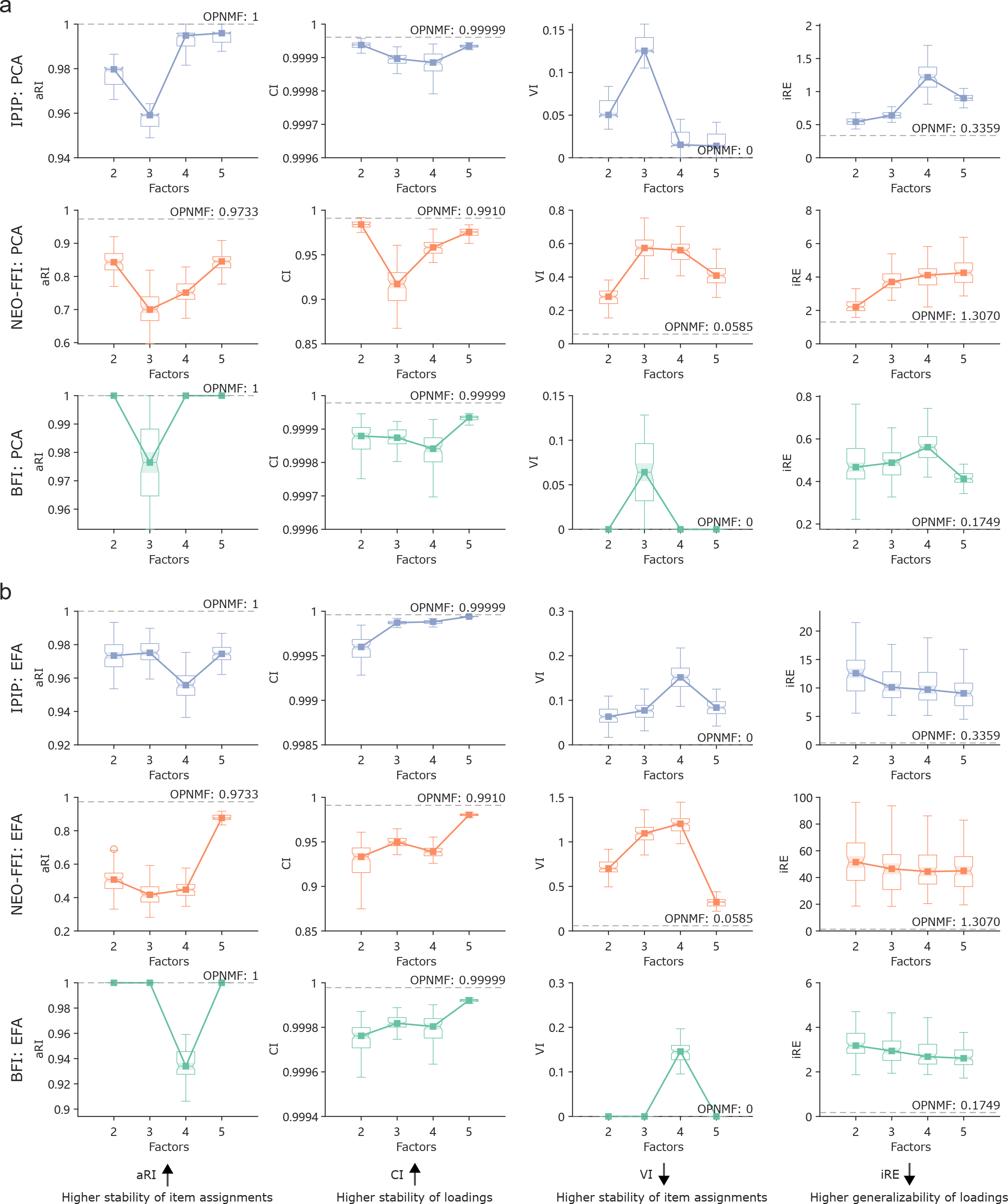


**Figure S6. Model evaluation in principal component analysis (PCA) and exploratory factor analysis (EFA).** The same analytical workflow and evaluation indices employed for OPNMF were also applied to assess factor decomposition using PCA (**a**) and EFA (**b**) across multiple inventories. PCA was conducted based on singular value decomposition, while EFA was performed using principal axis factoring with 100 iterations. Both solutions were subjected to oblique rotation. Evaluation indices derived from 1,000 rounds of five-fold cross-validation demonstrate robust item assignment consistency (higher aRI and lower VI) and strong similarity between loading coefficients (higher CI and lower iRE) across successive iterations of PCA/EFA-based decomposition. Box plots illustrate the median, lower and upper quartiles, as well as non-outlier maximum and minimum data points. The dashed line represents the decision value of OPNMF under the corresponding index (i.e., the median evaluation index of the optimal solution). Compared to EFA, PCA demonstrated superior performance in accommodating the two-factor model, especially with regard to the robustness of loading coefficients (i.e., CI and iRE).


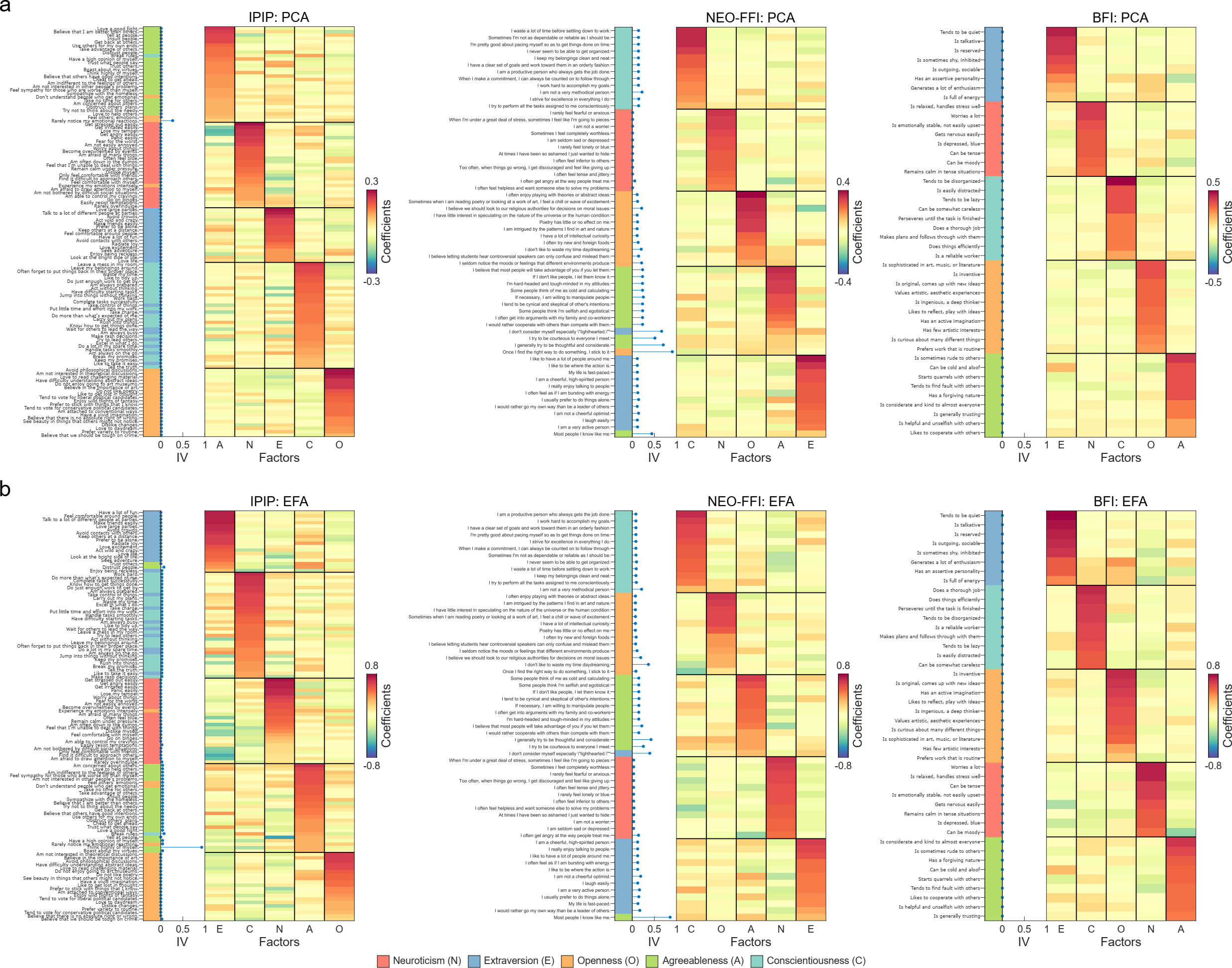


**Figure S7. Five-factor decomposition derived from PCA and EFA.** Personality trait decompositions based on PCA (**a**) and EFA (**b**) are shown in heatmaps. Items are categorized into the Big Five traits based on their maximum absolute loading coefficients across multiple inventories. Factors are represented by distinct colors, corresponding to the theoretical dimensions of the Big Five model, and are arranged vertically in descending order based on their loading coefficients. Item variability (IV) indicates the extent to which items may change their assignments during cross-validation.


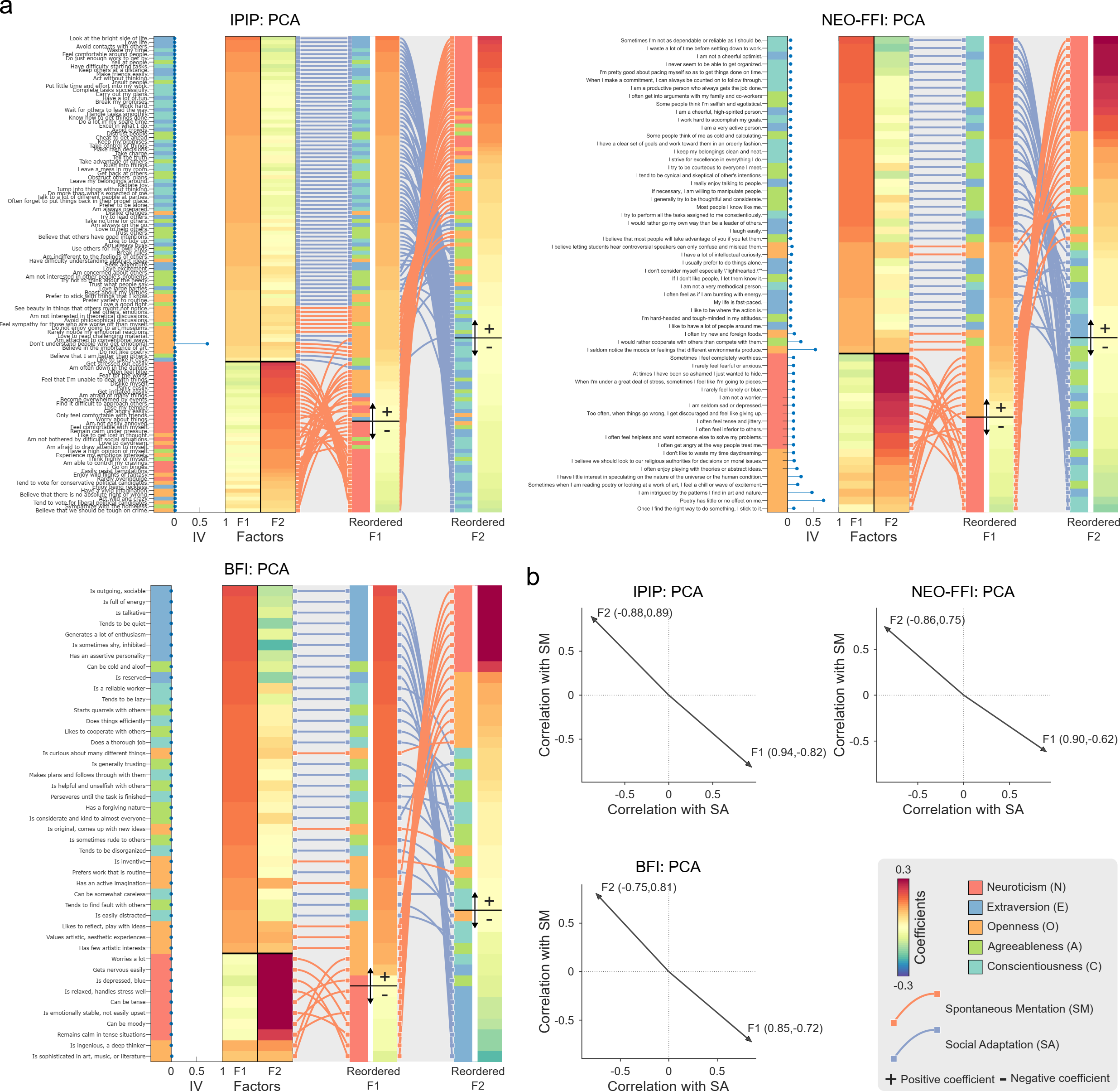


**Figure S8. Two-factor decomposition derived from PCA. a.** Heatmaps of two-factor decomposition across multiple inventories. In the left-panel heatmaps, items are categorized into two factors according to their maximum absolute loading coefficients. In the right-panel heatmaps, items within each factor are arranged in descending order according to their loading coefficients, thereby optimizing the visualization of positive covariance explained by each dimension. The alignment between PCA and OPNMF results reveals consistent personality trait covariations: Extraversion, Conscientiousness, and Agreeableness cluster together (Social Adaptation), while Neuroticism and Openness form another group (Spontaneous Mentation) based on observed positive covariance. PCA represents these as opposite poles of a single dimension, while OPNMF captures them as distinct factors with both methods confirming these factors as fundamental personality structures. **b.** Correlation between PCA-derived factor scores and OPNMF-derived factor scores. To elucidate the relationships between PCA and OPNMF decomposition results, PCA-derived factors were projected onto a two-dimensional space defined by Spontaneous Mentation and Social Adaptation through Pearson correlation coefficients. The vector coordinates represent the correlation patterns between PCA-derived and OPNMF-derived factor scores across these two dimensions. This analysis revealed that PCA-derived factors demonstrate robust correlations with their OPNMF-derived counterparts, capturing linear combinations of Spontaneous Mentation and Social Adaptation from opposing directional vectors. Notably, these directional projections of PCA-derived factors exhibit high consistency across diverse personality inventories.


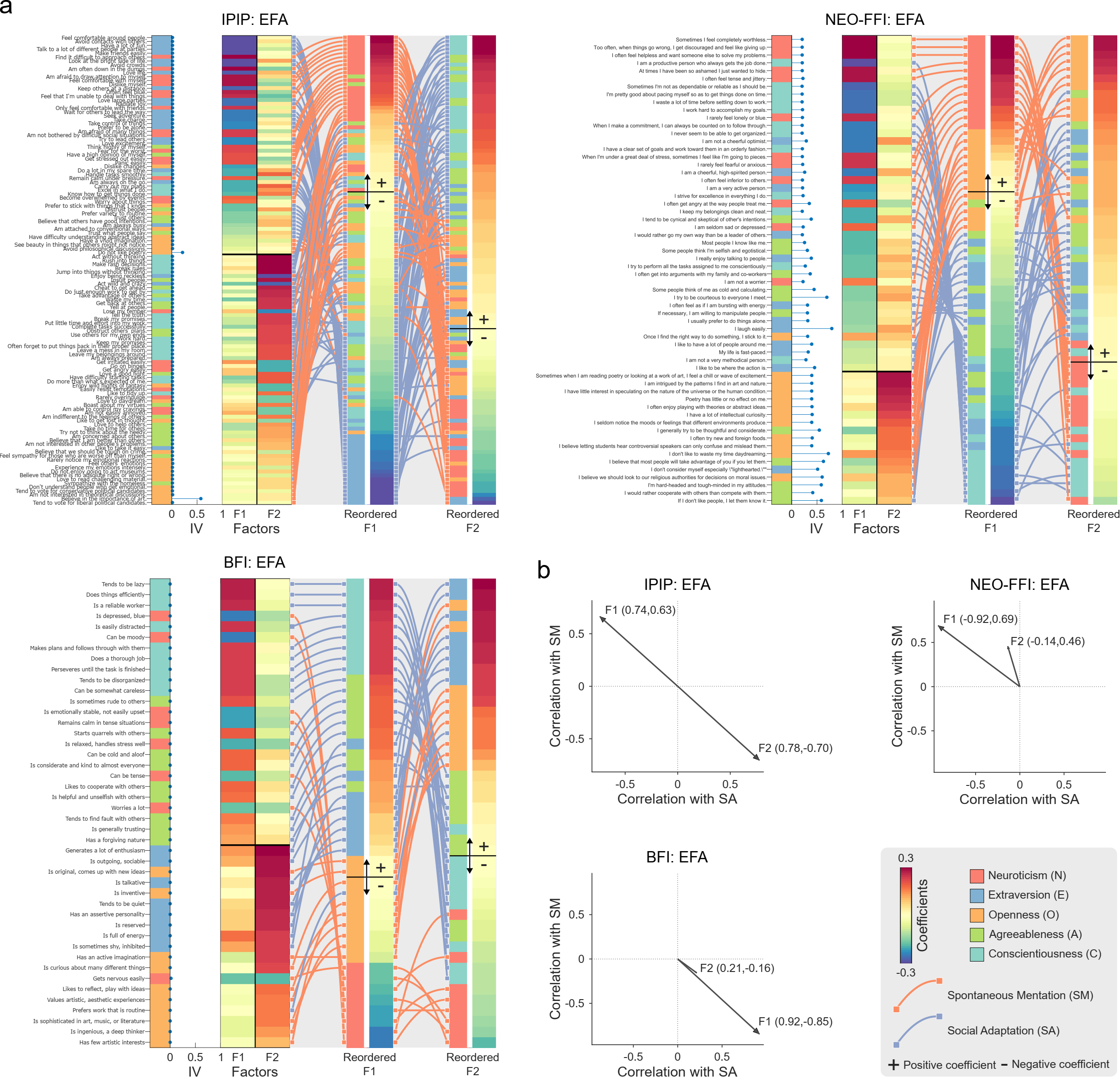


**Figure S9. Two-factor decomposition derived from EFA. a.** Heatmaps of two-factor decomposition across multiple inventories. In the left-panel heatmaps, items are categorized into two factors according to their maximum absolute loading coefficients. In the right-panel heatmaps, items within each factor are arranged in descending order according to their loading coefficients, thereby optimizing the visualization of positive covariance explained by each dimension. Compared to PCA, EFA places greater emphasis on the negative correlation between Neuroticism and the cluster formed by Agreeableness, Conscientiousness, and Extraversion. This leads traditional EFA-based methods to downplay the positive covariance between Neuroticism and Openness. However, when the loadings are arranged according to their directions, a consistent dimension can be observed that captures the positive covariation within Spontaneous Mentation and Social Adaptation, respectively. **b.** Correlation between EFA-derived factor scores and OPNMF-derived factor scores. To elucidate the relationships between EFA and OPNMF decomposition results, EFA-derived factors were projected onto a two-dimensional space defined by Spontaneous Mentation and Social Adaptation through Pearson correlation coefficients. The vector coordinates represent the correlational patterns between EFA-derived and OPNMF-derived factor scores across these two dimensions. Similar to PCA, the first dimension derived from EFA largely represents a strong linear combination of Spontaneous Mentation and Social Adaptation. However, the second dimension derived from EFA exhibits significant variation across different inventories.


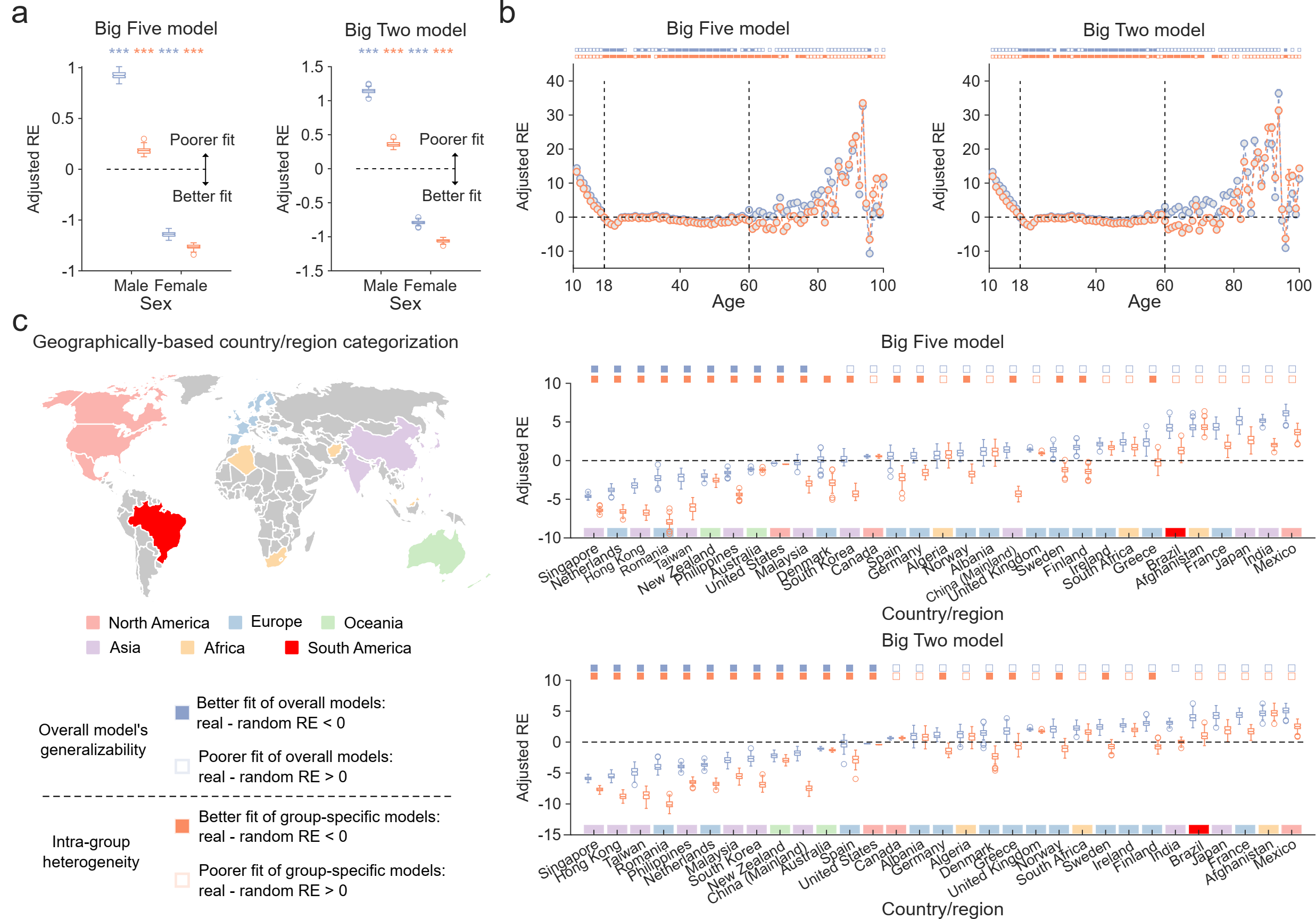


**Figure S10. Model generalizability and intra-group heterogeneity across population subgroups. a.** Gender-specific analysis of model performance. *** FDR, *q* < 0.001, two-tailed. **b.** Age-specific analysis of model performance. **c.** Culture-specific analysis of model performance. The blue dots correspond to the overall model’s generalizability, while the orange dots correspond to intra-group heterogeneity. Lower values correspond to better model fit, indicating improved generalizability or reduced intra-group heterogeneity. To maintain the reliability of analysis, only countries/regions with over 800 questionnaire responses are included. Here, countries/regions are labeled according to their geographical locations. In the above analyses, the overall model's generalizability is identified based on the reconstruction error (RE) when applying the overall model to various demographic and cultural subgroups. The RE value is adjusted by subtracting the value obtained with the actual group labels from the values obtained with 100 random group labels. Similarly, intra-group heterogeneity is identified by the adjusted RE when applying population-specific models to their respective subgroups. The data points in the figure represent the distribution of 100 adjusted RE values for both model generalizability and intra-group heterogeneity. The fit of the overall model or group-specific models within given subgroups can be determined by comparing the adjusted RE values to zero using a one-sample t-test. If the adjusted RE falls significantly below zero, it would indicate that the model's fit error is lower than what would be expected by chance alone. Conversely, an adjusted RE significantly above zero would indicate that the model's fit error exceeds what would be expected from random variation alone.


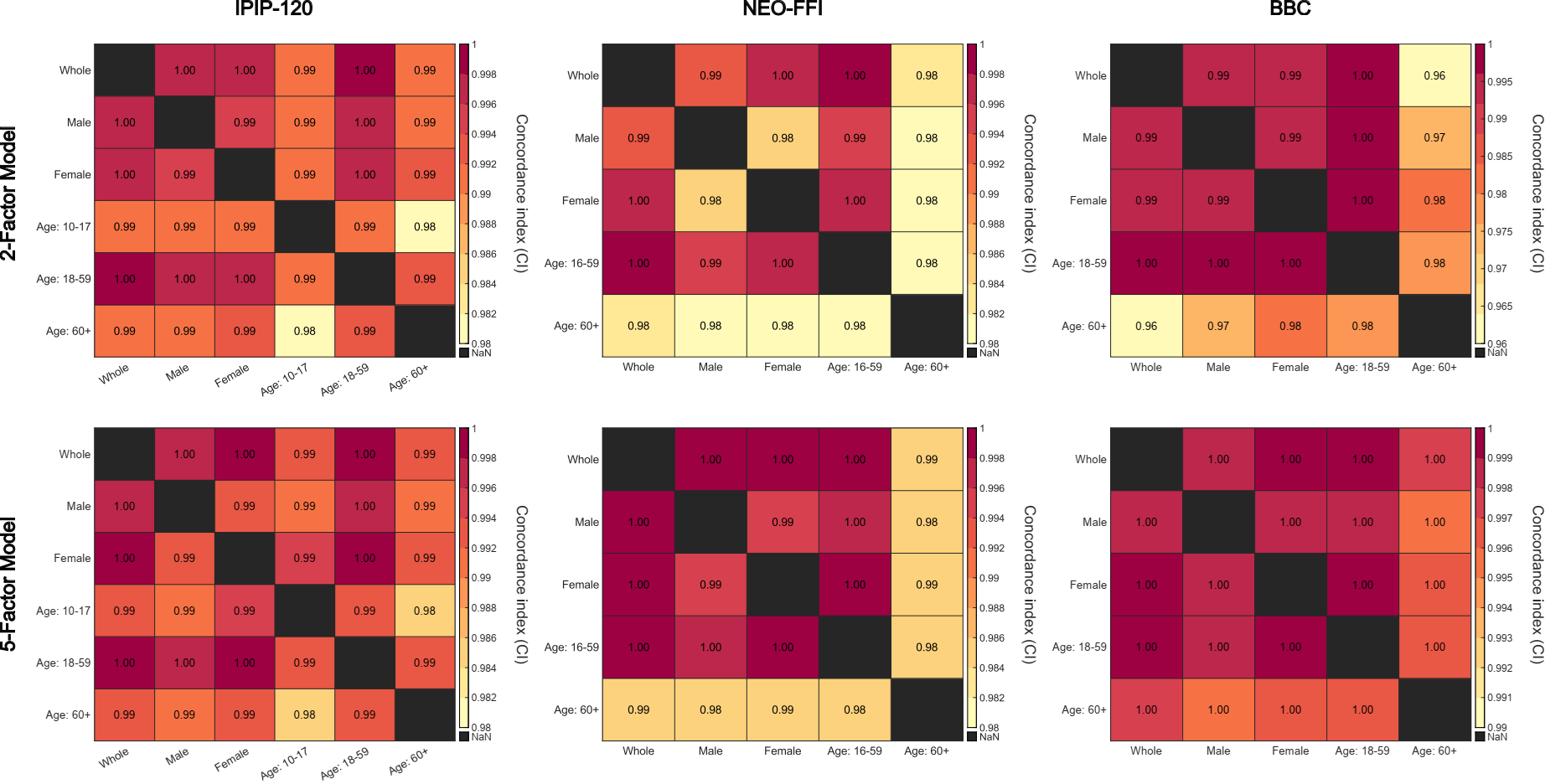


**Figure S11. Comparisons of factor decompositions tailored to various demographic subgroups.** The concordance index (CI) quantifies the degree of similarity between pairwise basis matrices (loading coefficients) across personality models derived from three independent datasets utilizing different five-factor model-based questionnaires (IPIP-120, NEO-FFI, and BBC-44). A CI value of 1 denotes perfect similarity between two models. The designations "sex" and "age" indicate models constructed using these specific demographic variables, while "Whole" refers to the model generated from the complete sample population.


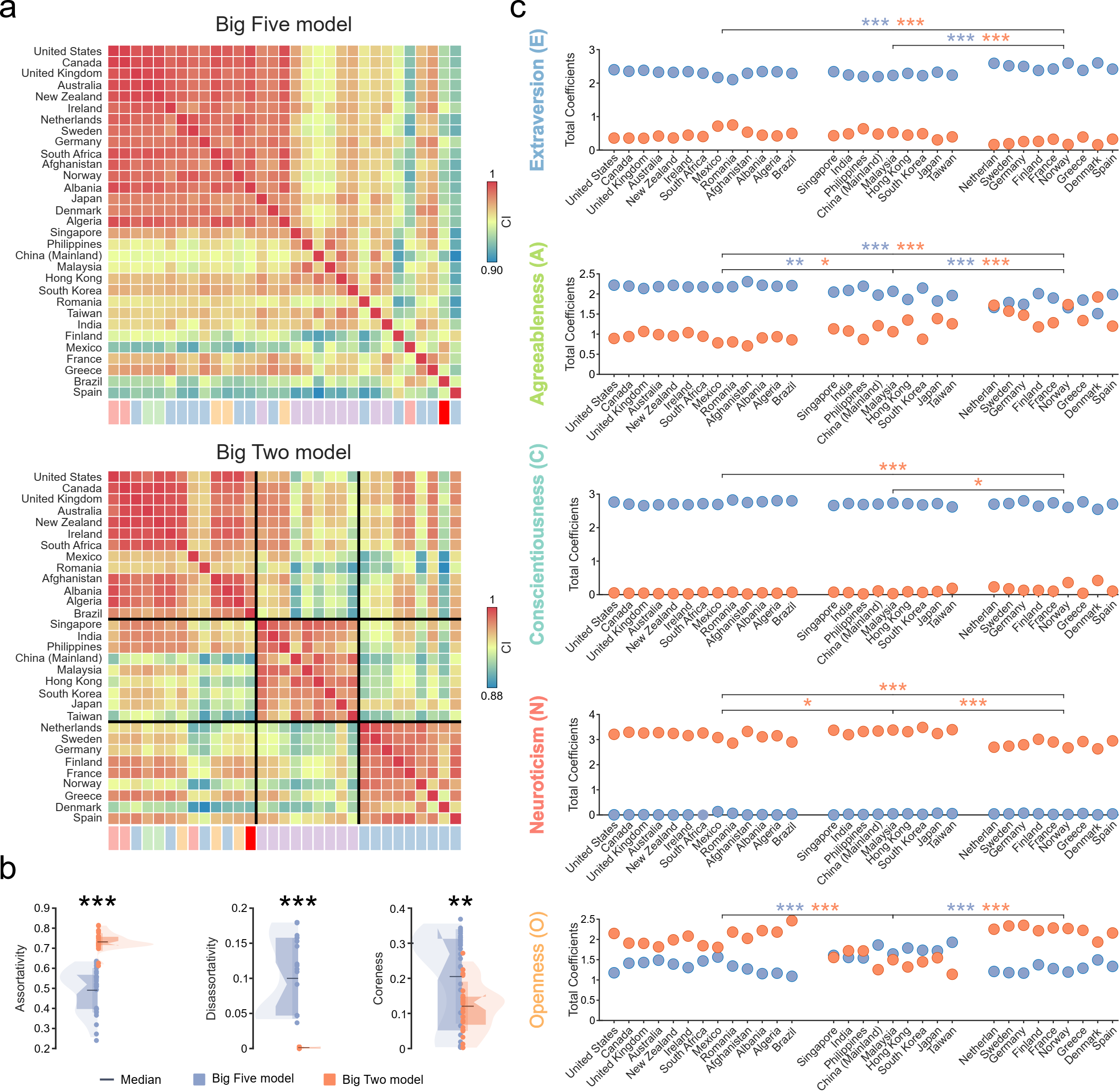


**Figure S12. The influence of cultural factors on personality structure. a.** Consistency of the models across subgroups identified by 31 countries/regions. Model consistency is measured using pairwise CI values ranging from 0 to 1. **b.** Assortative and non-assortative community structures of culturally-bound personality patterns in personality models. The association patterns of personality models differ between the Big Five and Big Two models. In comparison to the Big Five models, the Big Two models display higher assortativity (two-sample t-tests, *t* = 11.32, *p* < 0.001, two-tailed), lower disassortativity (*t* = -9.62, *p* < 0.001), and lower coreness (*t* = -3.11, *p* < 0.005) across different countries/regions (Supplementary methods). Consequently, the association patterns of the Big Five traits predominantly exhibit a non-assortative community structure, with personality models in different countries/regions converging towards network cores represented by countries in the core Anglosphere. However, the assortative community structure within Big Two traits highlights the segregation of personality networks, supporting distinct personality models within culturally-bound communities. Accordingly, the community detection on the Big Two traits divides the countries/regions of interest into three cultural spheres (delineated by black lines). Interestingly, this division is consistent with the geographic locations of different cultures, reflecting the influence of geography on personality shaping. The Asian cultural sphere exclusively consisted of Asian countries/regions, whereas the European cultural sphere exclusively encompassed European countries. Conversely, the Multicultural sphere included countries of interest from the Americas, Oceania, Africa, as well as several European countries. **c.** Varying contributions of five theoretical dimensions to the two Big Two traits across multiple subgroups. All modifications, except for those applied to Openness, are non-structural; in other words, they scarcely affect the factor structure or the allocation of items. The blue and orange dots represent Social Adaptation and Spontaneous Mentation, respectively. The X-axis represents models specific to various cultural subgroups, while the Y-axis represents the contribution of each theoretical dimension (determined by total OPNMF-derived item coefficients) to different Big Two models. Two-sample t-tests are employed to compare the differences between models across the three cultural spheres. * FDR, *q* < 0.05; ** FDR, *q* < 0.005; *** FDR, *q* < 0.001 (two-tailed).


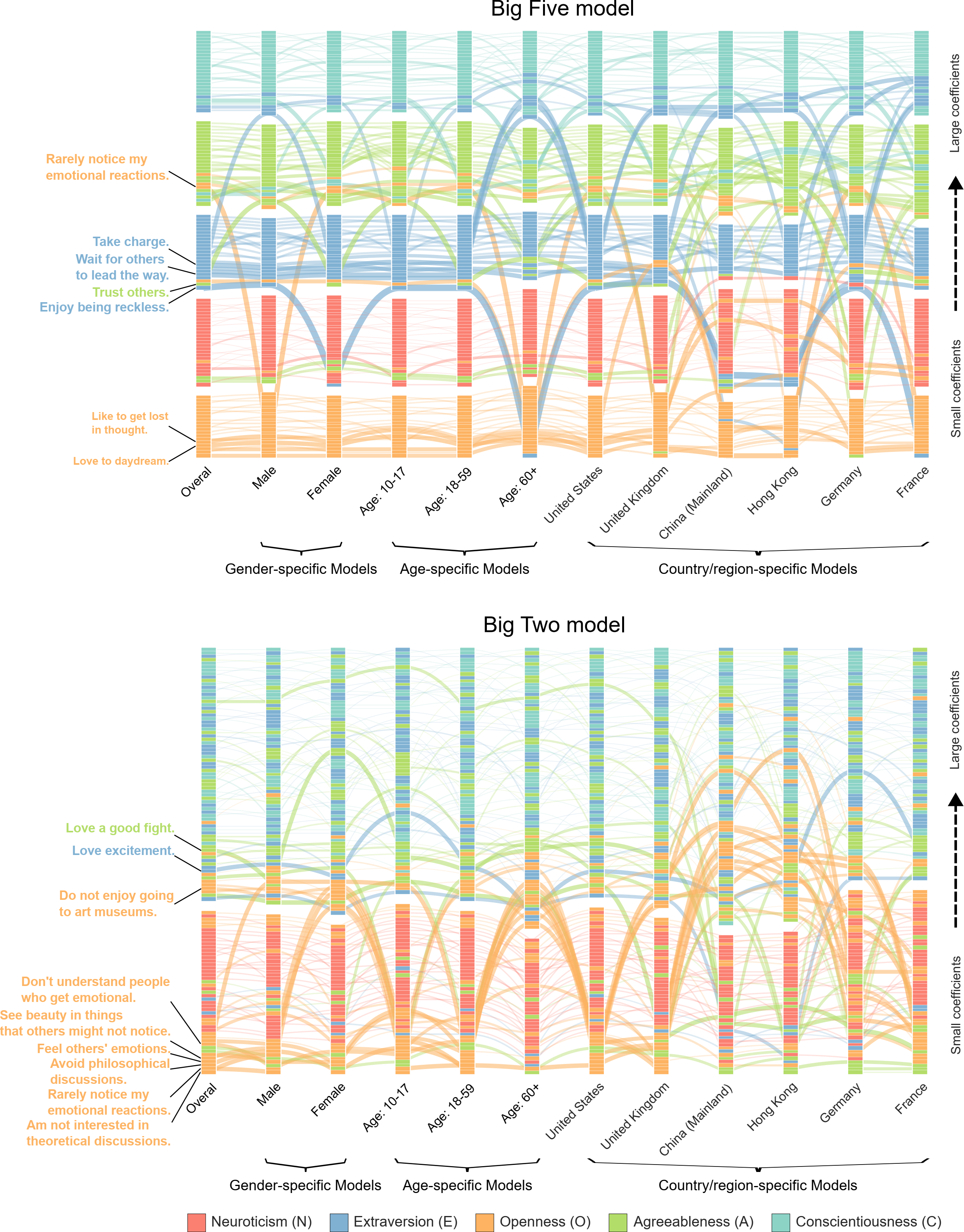


**Figure S13. Variations in item assignments across multiple subgroups.** Items are categorized into two-factor and five-factor models based on their maximum coefficients in the basis matrix derived from multiple subgroups. This method intuitively demonstrates the adjustments between models. Within each factor, items are color-coded according to their theoretical factors/dimensions in the IPIP-NEO, listed in descending order of their coefficients in the OPNMF basis matrix. To account for the large number of countries/regions examined, two representative countries/regions are selected from each cultural sphere. Items with a variability (measured by IV) exceeding two standard deviations are marked. Lines connect the same items across different subgroups, with thickness and transparency indicating item variability. Thicker and less transparent lines represent higher variability.


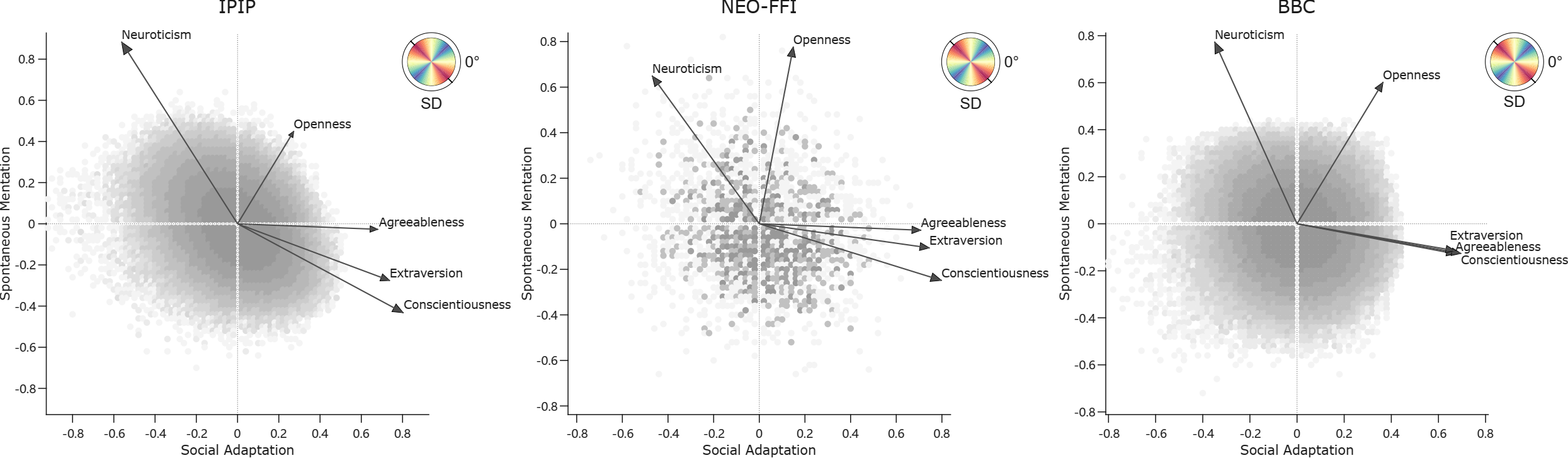


**Figure S14. The Big Two personality space.** A two-dimensional personality space comprising Social Adaptation and Spontaneous Mentation was constructed across three widely-used personality inventories. Participants' factor scores from the Big Two models were projected onto two-dimensional biplots. The density distribution of participants is represented by color intensity in the scatterplot, with darker hues indicating higher population density at specific coordinates. Vector characteristics such as direction and length represent the strength of each trait's correlation with the Big Two traits, quantified using Pearson's correlation coefficients between variable pairs. The angular relationship between vectors represents the correlation between traits: small angles indicate strong positive correlations, while opposing vectors denote negative correlations within the Big Two space. To standardize the visualization, individual data points (participants’ factor scores) are scaled by dividing each loading by the maximum absolute loading value and multiplying by the maximum coefficient length derived from Pearson's correlation coefficients. SD represents the standard deviation of the vector projection lengths in different directions, reflecting the variation of personality traits across various dimensions (ranging from blue to red, indicating increasing variation). The black line indicates the peak direction with the highest SD.


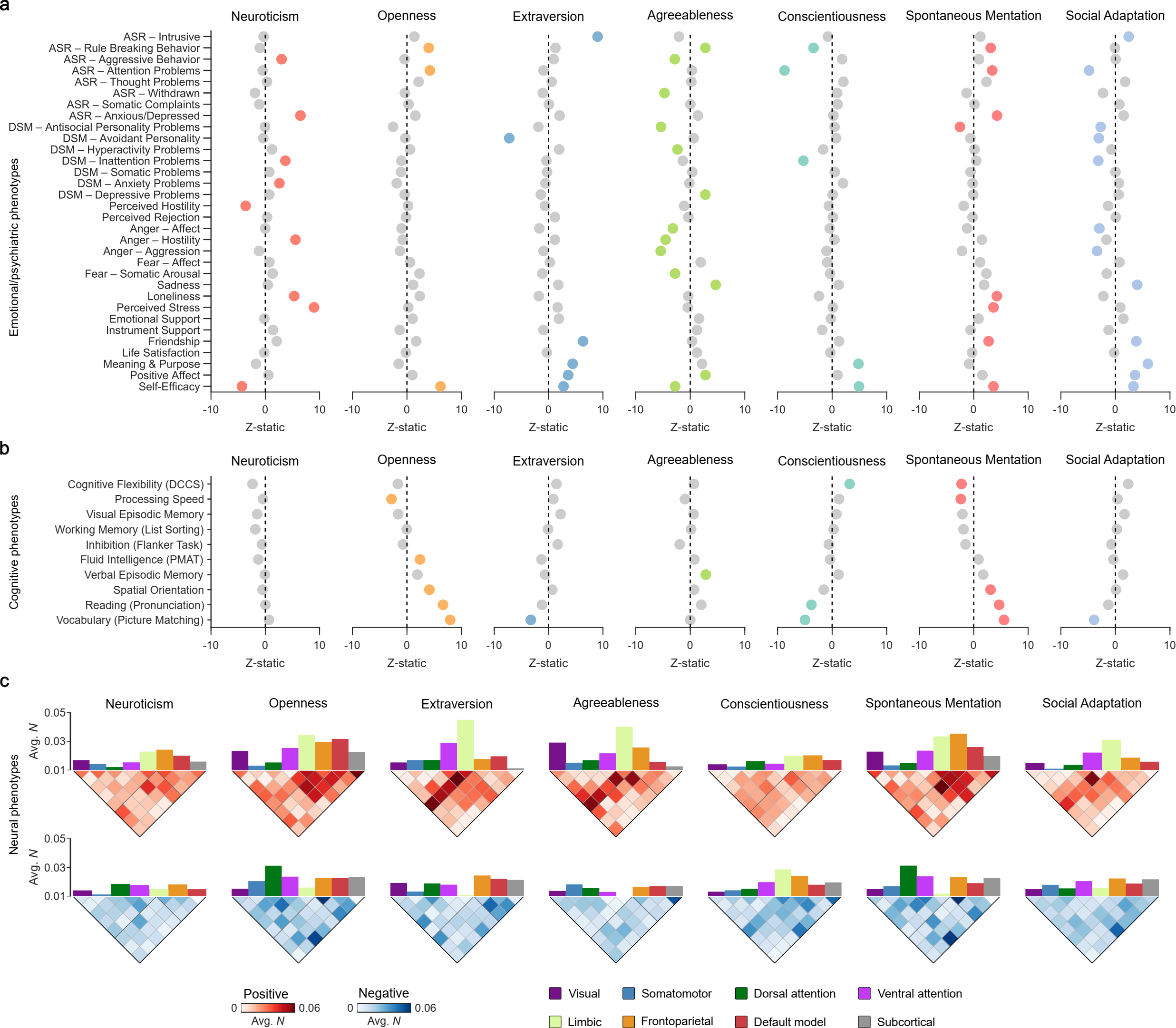


**Figure S15. Behavioural and neural phenotypic associations in the Big Five and Big Two dimensions. a.** Predictive weights of the emotional/psychiatric model. **b.** Predictive weights of the cognitive model. Gray circles indicate non-significant feature weights, whereas colored circles denote significance at FDR, *q* < 0.05 (two-tailed). **c.** Predictive weights of the functional connectivity model. Functional connectivity features (edges) are thresholded at uncorrected *p* < 0.05 (two-tailed). Significant features are displayed within each functional brain network and their interactions. Here, feature weights represent the cumulative number of significant edges. Edge counts are normalized at the network level by dividing by the total number of edges that exist within or between networks.


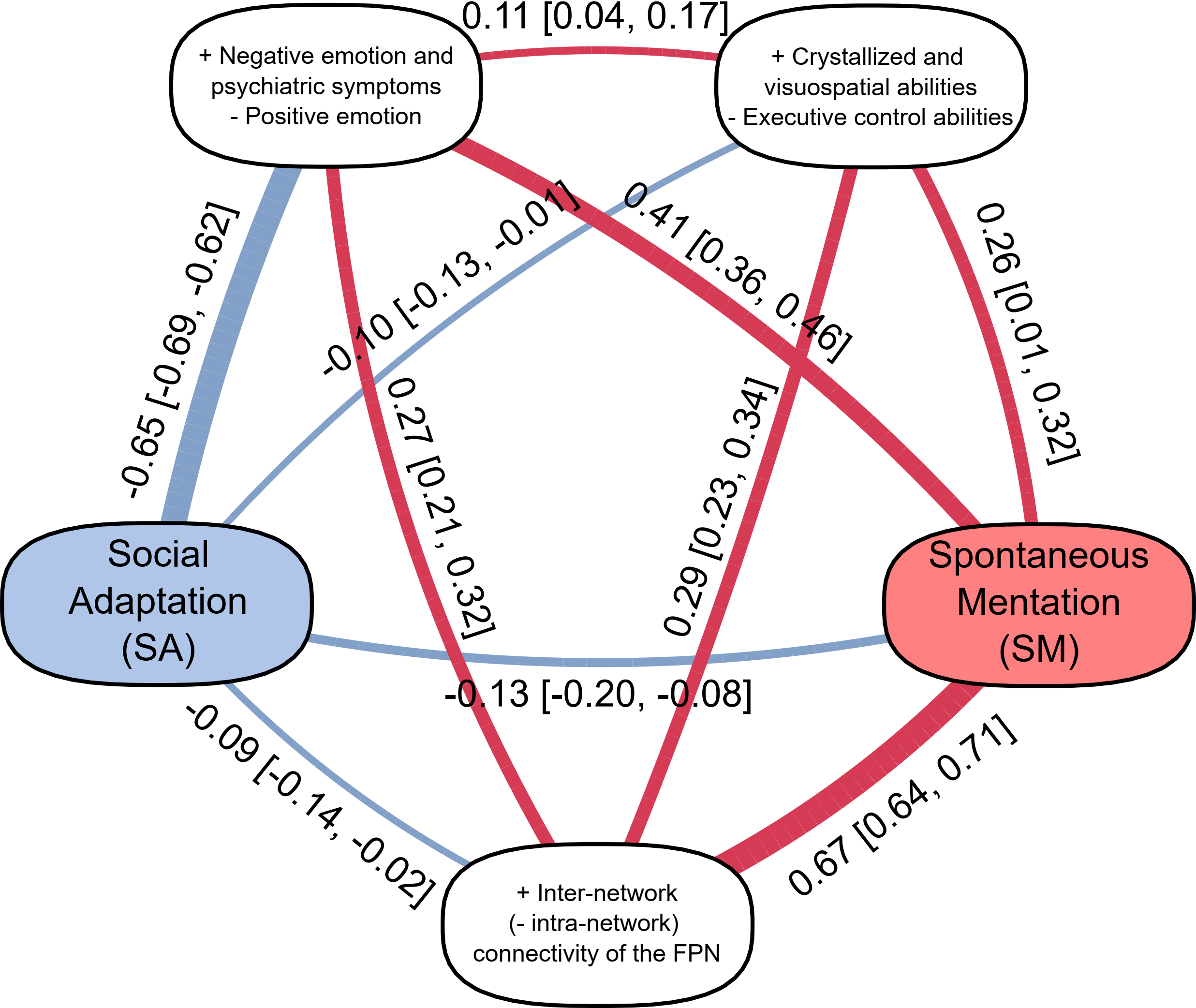


**Figure S16. Phenotypic associations between the Big Two dimensions and their signature response profiles.** Network representation of correlations between Social Adaptation (SA), Spontaneous Mentation (SM), and their associated signature response clusters. Edges depict Pearson correlations, annotated with bootstrap-based 95% confidence intervals. Red lines indicate positive associations and blue lines indicate negative associations, with line thickness proportional to the absolute correlation magnitude.


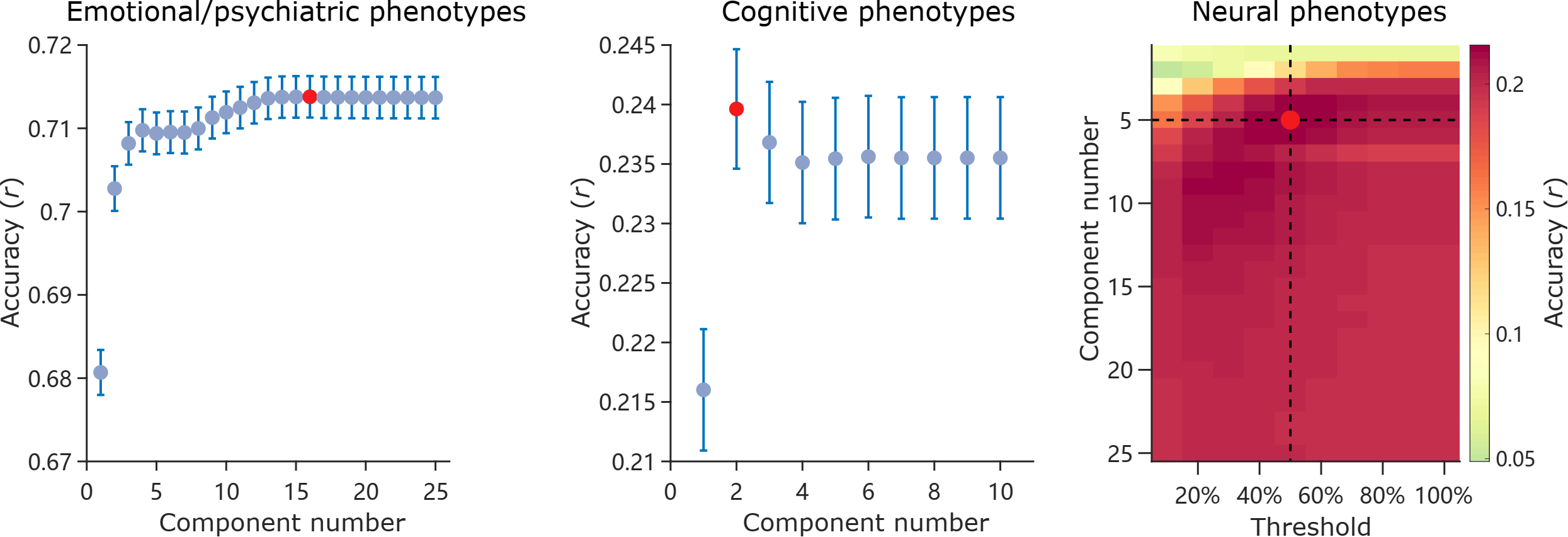


**Figure S17. Evaluation of parameters for the T-PLS models.** The figure illustrates the performance of the T-PLS model's parameters across different phenotypic data. The dots represent the mean prediction accuracy under different parameters across 30 cross-validation runs. The parameter with the highest mean value is selected as the optimal parameter (indicated by a red dot). The behavioural model is optimized solely for the number of components, while the neural model is optimized for both the number of components and feature sparsity.

# Supplementary Tables

**Table S1. The full set of model evaluations for the IPIP-NEO.**

**Table S2. The full set of model evaluations for the NEO-FFI and the NEO-PI-R.**

**Table S3. Stability of Big Two after ipsatization across three questionnaires**

| Questionnaire | CI | *r* (SA) | *r* (SM) |
| --- | --- | --- | --- |
| IPIP-120 | 0.98 | 0.97 | 0.95 |
| NEO-FFI | 0.99 | 0.99 | 0.97 |
| BFI | 0.98 | 0.97 | 0.94 |

**Note.** To evaluate the impact of acquiescence control, all items were ipsatized (i.e., within-participant mean-centered prior to reverse-scoring) and the Big Two model was re-estimated separately for each questionnaire. CI denotes the concordance index comparing loading similarity matrices derived from the ipsatized and original (non-ipsatized) solutions. *r*(SA) and *r*(SM) are Pearson correlations between the ipsatized and original item loadings, computed separately for each Big Two dimension. SA refers to Social Adaptation, and SM refers to Spontaneous Mentation. Correlations were calculated across items within each questionnaire.

# References

Aicher, C., Jacobs, A. Z., & Clauset, A. (2013). Adapting the stochastic block model to edge-weighted networks. *arXiv preprint arXiv:1305.5782*.

Aicher, C., Jacobs, A. Z., & Clauset, A. (2015). Learning latent block structure in weighted networks. *Journal of Complex Networks*, *3*(2), 221-248.

Avena-Koenigsberger, A., Goni, J., Solé, R., & Sporns, O. (2015). Network morphospace. *Journal of the Royal Society Interface*, *12*(103), 20140881.

Berry, M. W., Browne, M., Langville, A. N., Pauca, V. P., & Plemmons, R. J. (2007). Algorithms and applications for approximate nonnegative matrix factorization. *Computational statistics & data analysis*, *52*(1), 155-173.

Betzel, R. F., Bertolero, M. A., & Bassett, D. S. (2018). Non-assortative community structure in resting and task-evoked functional brain networks. *bioRxiv*, 355016.

Betzel, R. F., Medaglia, J. D., & Bassett, D. S. (2018). Diversity of meso-scale architecture in human and non-human connectomes. *Nature communications*, *9*(1), 346.

Boutsidis, C., & Gallopoulos, E. (2008). SVD based initialization: A head start for nonnegative matrix factorization. *Pattern recognition*, *41*(4), 1350-1362.

Camilleri, J., Eickhoff, S., Weis, S., Chen, J., Amunts, J., Sotiras, A., & Genon, S. (2021). A machine learning approach for the factorization of psychometric data with application to the Delis Kaplan Executive Function System. *Scientific reports*, *11*(1), 16896.

Chen, J., Patil, K. R., Weis, S., Sim, K., Nickl-Jockschat, T., Zhou, J., Aleman, A., Sommer, I. E., Liemburg, E. J., & Hoffstaedter, F. (2020). Neurobiological divergence of the positive and negative schizophrenia subtypes identified on a new factor structure of psychopathology using non-negative factorization: An international machine learning study. *Biological psychiatry*, *87*(3), 282-293.

Chen, J., Xiao, S., Zhang, P., Luo, K., Lian, D., & Liu, Z. (2024). Bge m3-embedding: Multi-lingual, multi-functionality, multi-granularity text embeddings through self-knowledge distillation. *arXiv preprint arXiv:2402.03216*.

Cheng, B., Chen, J., Königsberg, A., Mayer, C., Rimmele, L., Patil, K. R., Gerloff, C., Thomalla, G., & Eickhoff, S. B. (2023). Mapping the deficit dimension structure of the National Institutes of Health Stroke Scale. *Ebiomedicine*, *87*, 104425.

DeYoung, C. G., Quilty, L. C., & Peterson, J. B. (2007). Between facets and domains: 10 aspects of the Big Five. *Journal of personality and social psychology*, *93*(5), 880.

Dubois, J., Galdi, P., Han, Y., Paul, L. K., & Adolphs, R. (2018). Resting-state functional brain connectivity best predicts the personality dimension of openness to experience. *Personality neuroscience*, *1*, e6.

Fabrigar, L. R., Wegener, D. T., MacCallum, R. C., & Strahan, E. J. (1999). Evaluating the use of exploratory factor analysis in psychological research. *Psychological methods*, *4*(3), 272.

Guo, Y.-T., Li, Q.-Q., & Liang, C.-S. (2024). The rise of nonnegative matrix factorization: algorithms and applications. *Information Systems*, 102379.

Hubert, L., & Arabie, P. (1985). Comparing partitions. *Journal of classification*, *2*(1), 193-218.

Kohoutová, L., Heo, J., Cha, S., Lee, S., Moon, T., Wager, T. D., & Woo, C.-W. (2020). Toward a unified framework for interpreting machine-learning models in neuroimaging. *Nature protocols*, *15*(4), 1399-1435.

Lancichinetti, A., & Fortunato, S. (2012). Consensus clustering in complex networks. *Scientific reports*, *2*(1), 1-7.

Lee, D. D., & Seung, H. S. (1999). Learning the parts of objects by non-negative matrix factorization. *nature*, *401*(6755), 788-791.

Lee, S., Bradlow, E. T., & Kable, J. W. (2022). Fast construction of interpretable whole-brain decoders. *Cell Reports Methods*, *2*(6).

Meilă, M. (2003). Comparing clusterings by the variation of information. In *Learning theory and kernel machines* (pp. 173-187). Springer.

Raguideau, S., Plancade, S., Pons, N., Leclerc, M., & Laroche, B. (2016). Inferring aggregated functional traits from metagenomic data using constrained non-negative matrix factorization: Application to fiber degradation in the human gut microbiota. *PLoS computational biology*, *12*(12), e1005252.

Reimers, N., & Gurevych, I. (2019). Sentence-bert: Sentence embeddings using siamese bert-networks. *arXiv preprint arXiv:1908.10084*.

Sotiras, A., Resnick, S. M., & Davatzikos, C. (2015). Finding imaging patterns of structural covariance via non-negative matrix factorization. *Neuroimage*, *108*, 1-16.

Soto, C. J., & John, O. P. (2017). The next Big Five Inventory (BFI-2): Developing and assessing a hierarchical model with 15 facets to enhance bandwidth, fidelity, and predictive power. *Journal of personality and social psychology*, *113*(1), 117.

Steen, M., Hayasaka, S., Joyce, K., & Laurienti, P. (2011). Assessing the consistency of community structure in complex networks. *Physical Review E*, *84*(1), 016111.

Strus, W., & Cieciuch, J. (2021). Higher-order factors of the big six–similarities between big twos identified above the big five and the big six. *Personality and Individual differences*, *171*, 110544.

Wolf, T., Debut, L., Sanh, V., Chaumond, J., Delangue, C., Moi, A., Cistac, P., Rault, T., Louf, R., & Funtowicz, M. (2020). Transformers: State-of-the-art natural language processing. Proceedings of the 2020 conference on empirical methods in natural language processing: system demonstrations,

Yang, Z., & Oja, E. (2010). Linear and nonlinear projective nonnegative matrix factorization. *IEEE Transactions on Neural Networks*, *21*(5), 734-749.
